# Development of standard computerised adaptive test (CAT) settings for the EORTC CAT Core

**DOI:** 10.1007/s11136-023-03576-x

**Published:** 2024-01-17

**Authors:** Morten Aa. Petersen, Hugo Vachon, Johannes M. Giesinger, Mogens Groenvold, the European Organisation for Research, Treatment of Cancer (EORTC) Quality of Life Group

**Affiliations:** 1 Palliative Care Research Unit, Department of Geriatrics and Palliative Medicine GP, Bispebjerg and Frederiksberg Hospital, University of Copenhagen, Bispebjerg Bakke 23B, 2400 Copenhagen NV, Denmark; 2 Quality of Life Department, European Organisation for Research and Treatment of Cancer, Brussels, Belgium; 3 Department of Psychiatry, Psychotherapy, Psychosomatics, and Medical Psychology, Innsbruck Medical University, Innsbruck, Austria; 4 Department of Public Health, University of Copenhagen, Copenhagen, Denmark

**Keywords:** EORTC QLQ-C30, Item bank, CAT, IRT

## Abstract

**Aims:**

Computerised adaptive test (CAT) provides individualised patient reported outcome measurement while retaining direct comparability of scores across patients and studies. Optimal CAT measurement requires an appropriate CAT-setting, the set of criteria defining the CAT including start item, item selection criterion, and stop criterion. The European Organisation for Research and Treatment of Cancer (EORTC) CAT Core allows for assessing the 14 functional and symptom domains covered by the EORTC QLQ-C30 questionnaire. The aim was to present a general approach for selecting CAT-settings and to use this to develop a portfolio of standard settings for the EORTC CAT Core optimised for different purposes and populations.

**Methods:**

Using simulations, the measurement properties of CATs of different length and precision were evaluated and compared allowing for identifying the most suitable settings. All CATs were initiated with the most informative QLQ-C30 item. For each domain two fixed-length and two fixed-precision standard CATs were selected focusing on efficiency (brief version) and precision (long), respectively.

**Results:**

The brief fixed-length CATs included 3–5 items each while the long versions included 5–8 items. The fixed-precision CATs aimed for reliability of 0.65–0.95 (brief versions) and 0.85–0.98 (long versions), respectively. Median sample size savings using the CATs compared to the QLQ-C30 scales ranged 20%-31%, although savings varied considerably across the domains.

**Conclusion:**

The EORTC CAT Core standard settings simplify selection of relevant and appropriate CATs. The CATs prioritise either brevity and efficiency or precision, but all provide increased measurement precision and hence, reduced sample size requirements compared to the QLQ-C30 scales. The CATs may be used as they are or modified to accommodate specific requirements.

**Supplementary Information:**

The online version contains supplementary material available at 10.1007/s11136-023-03576-x.

## Introduction

Conventionally, standardised questionnaires ask the same set of items to all respondents to ensure comparability across patients and studies. This also applies for traditional patient reported outcome (PRO) measures (PROMs) like the European Organisation for Research and Treatment of Cancer (EORTC) quality of life questionnaire core 30 (the EORTC QLQ-C30, in the following referred to as QLQ-C30 [1, 2]). However, to include items relevant for patients with heterogeneous symptom levels, such traditional PROMs often include items of limited relevance for the individual patient. ‘Modern’ measurement approaches based on item response theory (IRT) calibrated item banks may reduce the number of such irrelevant items [3, 4]. One of the major advantages of IRT calibrated item banks is that scores based on any subset of items from a bank are directly comparable. This means that items may be selected to customise the instrument to each study or even to the individual patient while retaining full comparability of scores. Computerised adaptive tests (CATs) utilise this feature to tailor the item selection to the individual thereby optimising item relevance and measurement precision [5].

The QLQ-C30 is one of the most widely used health-related quality of life questionnaires for cancer patients [2]. It consists of 30 items covering 14 functional and symptom domains plus overall health/quality of life. To improve measurement of the functional and symptom domains, the EORTC Quality of Life Group (QLG) has developed the EORTC CAT Core instrument [6, 7]. The EORTC CAT Core includes 14 item banks enabling CAT-assessment of the same domains as included in the QLQ-C30. Each item bank comprises between 7 and 34 items, with a total of 260 items, and includes the items of the QLQ-C30 [6]. This ensures maximum backward compatibility with the QLQ-C30 while allowing for tailored, optimised assessment of these core domains. Evaluations have shown that the EORTC CAT Core often significantly increases measurement power and thereby reduces sample size requirements compared to the QLQ-C30 [6, 7].

In CAT-assessment, items are selected sequentially in real time. At each step of a CAT-assessment, a current score is estimated based on responses to the items asked in the previous steps. Among the not yet asked items, the most relevant and informative item for a patient at the estimated score level is selected and presented. This item selection is continued until a predefined stop criterion has been fulfilled. A CAT-assessment requires a set of predefined criteria determining how the assessment should proceed. As a minimum, this should specify a start item to initiate the assessment, a criterion for selecting subsequent items, and a stop criterion for when to terminate the assessment. Such a collection of preselected criteria constitutes the configuration or settings of the CAT. We will refer to it as the ‘CAT-setting’ or simply the setting. The potential advantages of CAT depend on the CAT-setting. A poorly selected setting may result in inefficient measurement with low power and/or unnecessary high response burden. On the contrary, a sensible CAT-setting should balance measurement precision and response burden in the way that is optimal for a given purpose. However, selecting a sensible CAT-setting requires detailed knowledge about the psychometric properties of the items and evaluations of the impact of various criteria (different start items, asking more/fewer items, …). Selecting an appropriate CAT-setting may be greatly simplified if a collection of ‘standard’ CAT-settings with known measurement properties, optimised for different populations and purposes, is available. Such standard settings may be used as they are or may be modified to fulfil study specific requirements.

The aim of the current study was twofold: (1) to provide a general approach for identifying a sensible setting for CAT-assessment and (2) to use the approach to generate a suggested collection of standard CAT-settings for the EORTC CAT Core which have optimal measurement properties for different purposes and populations. The EORTC QLG has previously developed a portfolio of standard short forms (i.e. static questionnaires composed of items from the item banks) [8]. For ease of use, in particular for the joint use of CATs and short forms, the approach for constructing standard CAT-settings will, when possible, mimic the approach applied for constructing standard short forms based on the EORTC CAT Core item banks.

## Methods

### The QLQ-C30

The five functional and nine symptom scales of the QLQ-C30 each includes between one and five items (see Table 1). All functional and symptom items have four response options: ‘not at all’, ‘a little’, ‘quite a bit’, and ‘very much’ and all but the five physical functioning items use a ‘during the past week’ timeframe. All QLQ-C30 scale scores are obtained by summating the item responses and transforming into a 0–100 score [9].

**Table 1:** Number of items in each EORTC CAT Core item bank (#CAT) and each corresponding QLQ-C30 scale (#C30), means and standard deviations (SDs) for the mild, moderate, and severe population, respectively, for each of the 14 domains

Domain	#CAT	#C30	Mild		Moderate		Severe	
			Mean	SD	Mean	SD	Mean	SD
Physical functioning	31	5	49	15	34	8	21	10
Role functioning	10	2	47	11	36	6	27	8
Emotional functioning	24	4	53	13	40	7	30	8
Cognitive functioning	34	2	49	10	38	7	28	7
Social functioning	13	2	49	10	38	5	30	7
Fatigue	34	3	46	12	59	7	70	9
Nausea and vomiting	19	2	61	13	76	10	90	10
Pain	16	2	48	11	60	6	68	6
Dyspnoea	32	1	53	11	64	5	71	6
Insomnia	8	1	50	7	58	5	66	6
Lack of appetite	7	1	55	12	67	6	75	6
Constipation	10	1	53	10	63	5	71	7
Diarrhoea	13	1	54	12	66	7	76	8
Financial difficulties	9	1	54	10	65	6	73	7

The mild population represents patients typically having ‘a little’ or less symptoms, the moderate population represents patients typically having ‘a little’ to ‘quite a bit’ symptoms and the severe population represent patients typically having at least ‘quite a bit’ symptoms. All EORTC CAT Core outcomes are scored on T-score metrics, scaled so that the European general population has a mean of 50 and a standard deviation of 10. Functional scores > 50 reflect better functioning than the average general population; symptom scores > 50 reflect more symptoms than the average general population

### The EORTC CAT Core

The EORTC CAT Core includes 14 item banks covering the functional and symptom domains of the QLQ-C30 questionnaire [6] (additional items have not been developed for the overall health/quality of life domain [10]). Each item bank includes the QLQ-C30 item(s) of the particular domain supplemented with additional items, all using the same timeframe and response options. The number of items in each item bank and corresponding QLQ-C30 scale is shown in Table 1. All measures based on the EORTC CAT Core are scored on T-score metrics, scaled so that the European general population has a mean of 50 and a standard deviation of 10 [11]. This means that scores > 50 for functional domains reflect better functioning than the average of the European general population while for symptom domains scores > 50 reflect more symptoms than the average general population.

### CAT-settings

To identify the optimal CAT-settings and thereby select the standard CAT-settings, various combinations of start item, item selection criteria, and stop criteria were evaluated. We aimed to balance measurement precision and efficiency (i.e. low response time and burden) so that the standard settings provided high precision without burdening patients unduly. For each of the 14 domains, CAT-settings were developed to optimise the assessment of three patient populations with different levels of symptoms. For each population two fixed-length and two fixed-precision standard CATs were selected focusing on efficiency (brief version) and precision (long version), respectively (see below for details).

For all standard CAT-settings expected a posteriori (EAP) was used to estimate the domain scores [12].

### Target populations

We defined three target populations for each domain representing patients predominantly having mild, moderate, and severe symptoms, respectively. These were theoretical populations; the study did not include real-world data. The target populations were the same as used for the development of standard short forms and a detailed description of the populations is provided in [8]. The means of the three target populations were defined from the QLQ-C30 items. The mean of the mild population was the average score one would obtain if answering ‘not at all’ or ‘a little’, respectively, to the QLQ-C30 items of the domain. That is, the mild population represents patients typically having ‘a little’ or less symptoms. The two other populations were defined in a similar way so that the moderate population represents patients typically having ‘a little’ to ‘quite a bit’ symptoms, while the severe population represent patients typically having at least ‘quite a bit’ symptoms. Means and standard deviations for the three target populations across the 14 domains are provided in Table 1. These target populations can be used to select the a priori most relevant CAT-setting for a particular study. For example, for a study in cancer survivors, if expecting the survivors to typically have limited symptoms/problems, CAT-settings targeting the mild population will be particularly relevant.

### Start item

To ensure a direct link to the QLQ-C30, all standard CAT-settings were required to start with a QLQ-C30 item. For the eight domains including more than one QLQ-C30 item, we used the QLQ-C30 item providing the highest average amount of (Fisher) information [13] for the population in focus. To evaluate whether the requirement to start with a QLQ-C30 item affected the measurement properties of the CATs, the suggested standard CAT-settings were compared to CATs starting with the most informative item in the item bank (when this was not a QLQ-C30 item) while retaining all other criteria of the setting.

### Item selection

For all CAT-settings, the item providing the maximum Fisher information at the current domain score estimate was selected and presented in each step of the CAT. This is the most commonly applied selection rule [5, 14]. Some domains cover 2–4 content subdomains. For example, physical functioning covers subdomains concerning lifting/carrying, walking/moving, mobility (unspecified), and selfcare. Emotional functioning also includes three subdomains while role, cognitive and social functioning, and fatigue, nausea and vomiting, and pain includes two subdomains. The rest of the item banks covers one subdomain only. To ensure reasonable coverage of all subdomains it was required that at least one item was asked from each subdomain. This was achieved by selecting in each step of the CAT the best item among the subdomains not yet covered. When all subdomains were covered, i.e. one item had been asked from each subdomain, selection was free among all subdomains. To evaluate the possible impact on measurement properties of this content coverage requirement, the standard CAT-settings were compared to similar CATs without this requirement.

### Stop criteria

Two types of CATs were evaluated, ‘fixed-length’ and ‘fixed-precision’. In fixed-length CAT the assessment is terminated when a prespecified number of items has been asked, i.e. all patients are asked the same number of items. Fixed-length CATs asking up to 12 items were evaluated. In fixed-precision CATs, assessment is terminated when a prespecified level of precision (reliability) has been reached, i.e. patients may be asked different numbers of items. To ensure content coverage CATs were required to ask a minimum of one item per subdomain and hence, the minimum number of items that could be asked with a fixed-precision CAT was equal to the number of subdomains (between one and four items per domain). The maximum number of items to ask was set to eight or 12 items, respectively (i.e. two variants were evaluated). Fixed-precision CATs aiming for the following reliabilities were evaluated: 0.65, 0.70, …, 0.95, 0.98. Note that the aimed and actually attained reliability may differ as not all patients may attain the aimed level (particularly for high levels of reliability), and some may attain higher reliability (particularly for low levels of reliability). When in the following referring to reliability of the fixed-precision CATs this refers to the aimed reliability not the actually attained. The reliability is defined as the ratio of the true domain score variance and the observed score variance and estimated from the information function [15].

Generally, for the brief standard CAT-settings low response burden was prioritised while for the long versions measurement precision was in focus. For all settings, the choice of length/precision was a balancing of measurement precision and response burden. Hence, even though asking additional items always provide some extra information this may be so limited that also for the long version it was deemed unnecessary compared to the burden of answering additional items.

Patients answering ‘not at all’ symptoms/problems to the initial items likely do not have the symptom being measured. Hence, asking such patients many items often provide limited information and may be tiresome for the patients. To avoid this, a CAT-assessment can be stopped if the first X items are answered ‘not at all’. The impact on measurement properties and number of items asked when adding this criterion to the standard CAT-settings was evaluated. The effect of stopping if the first two items were answered ‘not at all’ was evaluated for all domains except physical functioning, emotional functioning, and fatigue for which four, three, and three items, respectively should be answered ‘not at all’ so that items from all subdomains were asked before stopping.

### Evaluation of CAT-settings

The following evaluations were conducted for each domain. At each of 161 equidistant score points across the range of possible domain scores (i.e. typically across 10–90 with increments of 0.5), 200 sets of responses to the items in the bank were generated using Monte Carlo simulation producing a total of 200 × 161 sets of items response. Using physical functioning and a score of 10 as example the simulation of the response sets was as follows: for each of the 31 physical functioning items the conditional probability of responding to each of the four response options when having a physical functioning score of 10 was estimated using the calibrated IRT model for the item bank. Based on these probabilities a random response was selected. For each set of responses, CAT-assessments using the various CAT-settings were simulated. That is, for a set of simulated response, first the response to the start item was used to produce an initial score estimate and from this the item estimated as most informative was selected. The simulated response to this item was then used to update the score estimate and select the next item. This selection was continued until the stop criterion had been reached. When reached, the final CAT-based score estimate was saved and compared to the simulated ‘true’ score (10 in the example). For each of the three target populations described above, the median difference (with lower and upper quartiles) between estimated scores and true scores were calculated, weighting the results from each score point by the density function of the specific target population. For the fixed-precision settings the weighted average number of items asked was also calculated.

A second Monte Carlo simulation study was conducted to evaluate the relative power of the CAT-settings to detect group differences. In each simulation, two groups of random size between 50 and 250 were sampled. Scores for one group was sampled randomly from the target population of focus and the other from a population whose mean differed randomly from the mean of the target population corresponding to an effect size difference between 0.2 and 0.5. This was done for each domain and each target population (mild, moderate, and severe symptoms), respectively. For example, for the population having mild physical problems, 100 physical functioning scores could be sampled from a normal distribution with mean = 49 forming one group and 100 could be sample from a normal distribution with mean = 44 forming the second group. Based on these sampled ‘true’ scores, item responses and CAT-assessments were simulated using a similar procedure as described above, producing CAT based estimated scores along with the standard sum scores based on the QLQ-C30 items [9]. Two-sample t-test sizes for comparing the two groups were calculated based on the CAT scores and the QLQ-C30 sum scores, respectively. To assess the relative validity (RV) of each CAT-setting compared to the QLQ-C30 sum scales the ratio of the test sizes based on the two scoring approaches was calculated:

An RV > 1 indicates higher measurement precision of the CAT than the QLQ-C30 scale [16]. The median relative validity across 1000 simulations was calculated and from this the median relative sample size requirement of the CATs compared to the QLQ-C30 scale were estimated [17].

All analyses and simulations were conducted using SAS Enterprise Guide 7.15. SAS code for conducting the CAT simulations used for evaluating the measurement properties is available in the Supplemental material.

## Results

We illustrate the procedure for identifying the optimal CAT-settings by describing in detail the procedure for a population with mild physical function problems. The identification of all other standard CAT-settings followed a similar procedure.

### Physical functioning

The physical functioning CAT item bank includes 31 items of which five derive from the QLQ-C30. The average information provided by each of the five QLQ-C30 items for a target population having mild physical problems is shown in Table 2. Of the five QLQ-C30 items, the item “Do you have any trouble taking a long walk?” was the most informative. Hence, this item was selected as start item for the CAT-settings for this population.

**Table 2:** Average information provided by each of the five QLQ-C30 physical functioning items and the most informative item in the item bank for a population with mild physical problems

Item text	Average information^a^
Do you have any trouble doing strenuous activities, like carrying a heavy shopping bag or a suitcase? (QLQ-C30)	0.015
Do you have any trouble taking a long walk? (QLQ-C30)	0.017
Do you have any trouble taking a short walk outside of the house? (QLQ-C30)	0.009
Do you need to stay in bed or a chair during the day? (QLQ-C30)	0.003
Do you need help with eating, dressing, washing yourself or using the toilet? (QLQ-C30)	0.005
Do you have any trouble carrying a heavy bag upstairs?	0.022

^a^Weighted average Fisher information across the score range weighted by the population density function. That is, it is the average information provided by each item for the target population (patients having mild physical problems)

All fixed-precision CATs were evaluated allowing a maximum of eight or 12 items, respectively. It was mainly those with extreme scores who were asked additional items when the maximum was increased from eight to 12 items. However, at the extreme scores limited information was available and hence, limited additional information was obtained. Setting the maximum to 12 instead of eight items only increased the reliability by 1% or less, i.e. the two versions resulted in highly similar reliabilities (details not shown). Therefore, to limit the response burden it seemed preferable to use a maximum of eight items. Similar trivial differences in reliability for the two versions were observed for all other domains. Therefore, all fixed-precision standard CATs ask a maximum of eight items.

**Fig. 1:**
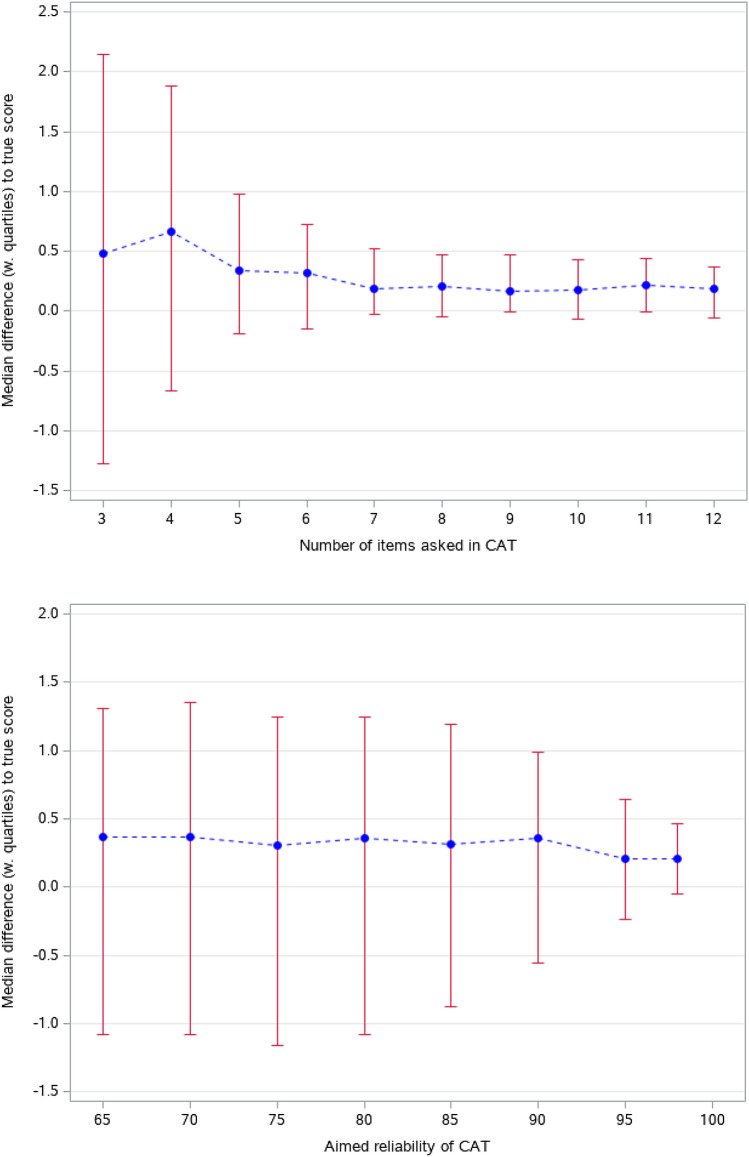
Median difference between CAT estimated scores and true physical functioning scores with lower and upper quartiles (25th and 75th percentiles) for a population with mild physical problems. Top plot: fixed-length CATs, bottom plot: fixed-precision CATs

**Fig. 2:**
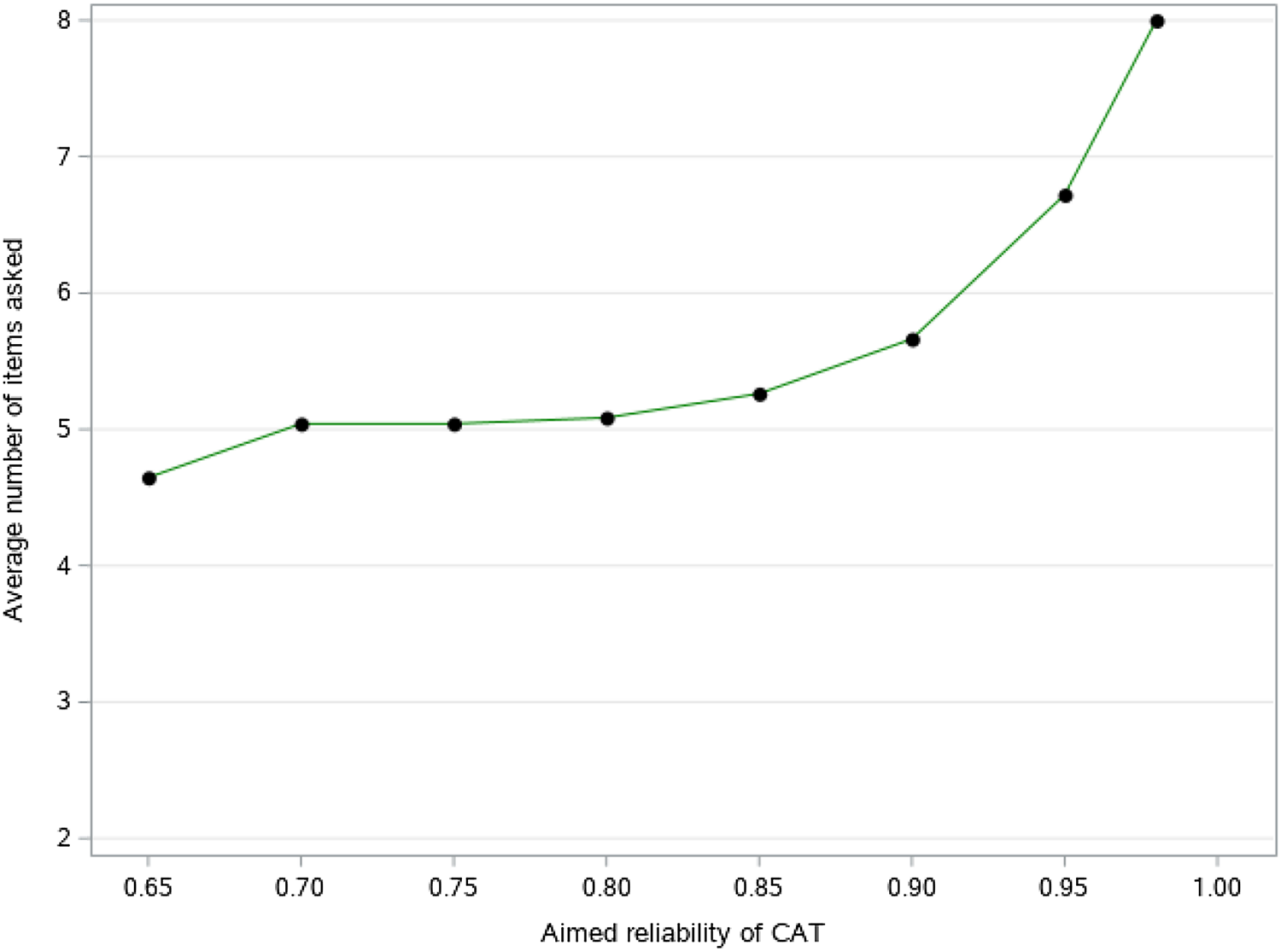
Average number of items asked with fixed-precision CATs for a population with mild physical problems (maximum number of items asked set to eight)

Figure 1 shows the median differences between CAT estimated physical functioning scores and true scores with lower and upper quartiles. The first plot presents results for fixed-length CATs asking 3–12 items. The median differences were small, all < 1 (i.e. < 0.1SD), however, the interquartile ranges (upper–lower quartile, IQRs) were markedly wider for the shortest CATs, particularly when asking < 5 items. The additional reductions in IQRs when asking more than seven items seemed trivial (< 0.05 per extra item). The fixed-precision CATs aiming for reliabilities 0.65–0.85 resulted in similar results. Inspecting Fig. 2 shows that these CATs also asked highly similar number of items reflecting that for many, reliability > 0.85 was obtained with four items. Aiming for higher reliabilities reduced IQRs. Note that reliability ≥ 0.98 was not attained in any cases with ≤ 8 items (median obtained reliability = 0.96 for this CAT), hence, all these CATs asked eight items.

**Fig. 3:**
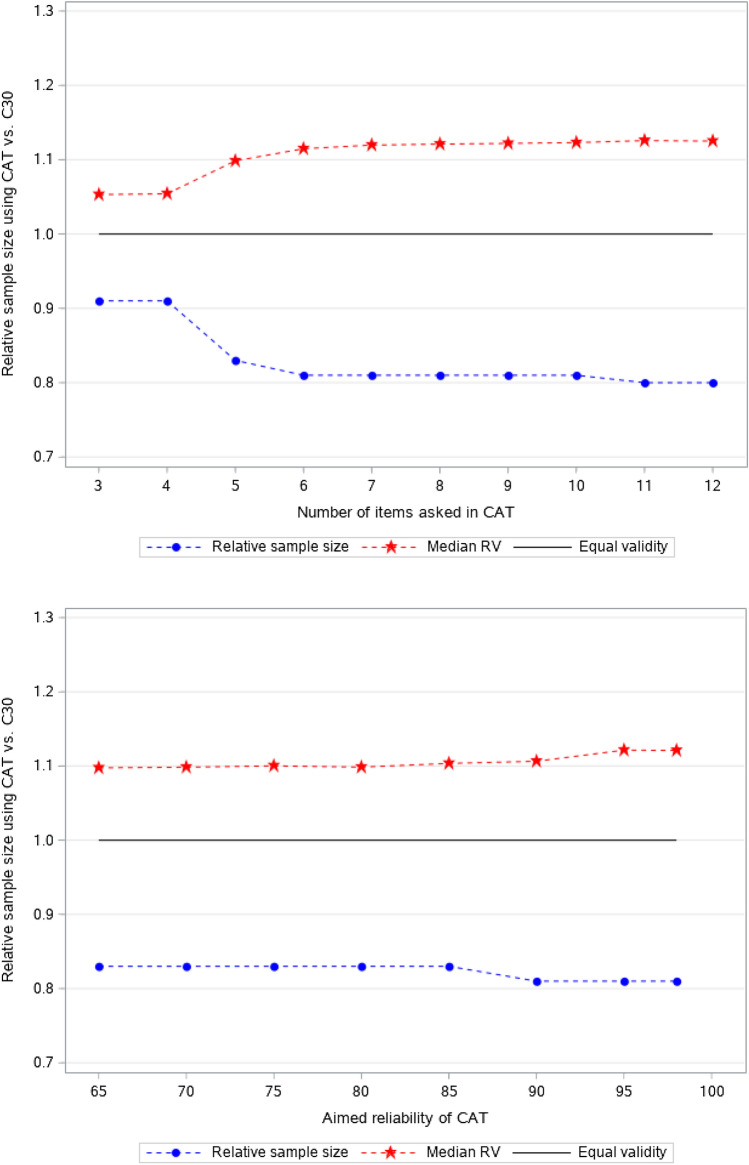
Relative validity and relative sample size required using different fixed-length (top plot) and fixed-precision (bottom plot) physical functioning CATs, respectively, compared to using the QLQ-C30 physical functioning scale for a population with mild physical problems

Figure 3 shows the median relative validities and relative sample size requirements of the different CATs compared to using the 5-item QLQ-C30 physical functioning scale. All relative validities were > 1 indicating generally higher power of the CATs compared to the QLQ-C30 physical functioning scale. The estimated relative samples using the CATs were 80–90% of the sample required using the QLQ-C30 scale, i.e. 10–20% smaller samples may be collected when using the CATs compared to using the QLQ-C30 scale without reducing the power to detect group differences. For fixed-length CATs, the sample size requirements decreased (i.e. power increased) with increasing number of items asked, up to six items. Asking more items only seemed to increase savings trivially (< 2% if asking more than six items). The fixed-precision CATs evaluated resulted in similar estimated relative sample size requirements only increasing trivially with increasing precision.

Taken together, the evaluations indicated that asking fewer than five items often resulted in large deviations between estimated and true scores, while asking more than seven items seemed to have limited impact on measurement precision and power. Therefore, the suggested fixed-length CATs for patient populations typically having mild physical problems ask five (brief) and seven (long) items, respectively. For fixed-precision CATs the best balance between efficiency and precision seemed to be attained for reliabilities of 0.90 (brief) and 0.95 (long), respectively.

Starting with the most informative item in the bank instead of the QLQ-C30 ‘long walk’ item (see Table 2) and not requiring content coverage had only trivial impact on precision and power for the CATs. Stopping the CATs if the first four items were answered ‘not at all’ reduced the number of items asked with about one item on average (range 0–4 items) but this also reduced the expected sample size savings with 5–8%. If efficiency (low response time and burden) is in focus, this stopping rule may be added to the standard CATs.

### Summary for all domains

Using similar approaches and arguments as presented above, CAT-settings across the 14 domains were evaluated and optimal CAT-settings identified. Supplemental material (Online Resource 1) shows the start items and the relative validity and sample size requirements of CATs of different length and precision compared to the QLQ-C30 for all domains (analogous to the information for physical functioning in Fig. 3). Further details may be obtained by contacting the first author or the EORTC QLG (https://qol.eortc.org/cat/). Table 3 presents the number of items/reliability of the fixed-length/fixed-precision standard CAT-settings. In this table, the estimated relative sample size savings compared to using the QLQ-C30 scales are also provided. The brief fixed-length CATs ask 3–5 items with a median of four items while the long versions ask 5–8 items with a median of six items. The selected level of reliability for the brief fixed-precision CATs varied between 0.65 and 0.95 (median 0.90) while for the long versions the reliability was 0.85–0.98 (median 0.95). The estimated savings in sample size requirements varied markedly across populations and domains from 3% (moderate physical functioning and mild pain) to 52% (moderate nausea and vomiting) with median sample savings across domains ranging 20% to 31%.

**Table 3:** Identified fixed-length (FL) and fixed-precision (FP) standard CAT-settings for populations with mild, moderate, and severe symptoms, respectively. The table shows the number of items (#) for fixed-length CATs and reliability (rel.) for fixed-precision standard CATs, respectively, with the estimated savings in sample size requirements compared to the QLQ-C30 scales (saving)

Domain		Mild, brief		Mild, long		Moderate, brief		Moderate, long		Severe, brief		Severe, long	
		FL	FP	FL	FP	FL	FP	FL	FP	FL	FP	FL	FP
Physical functioning	#/rel Saving	5 17%	0.90 19%	7 19%	0.95 19%	5 3%	0.95 8%	8 11%	0.98 12%	5 14%	0.95 14%	8 18%	0.98 17%
Role functioning	#/rel Saving	4 20%	0.85 19%	6 22%	0.95 23%	4 22%	0.90 16%	6 27%	0.95 23%	4 19%	0.85 12%	6 23%	0.95 23%
Emotional functioning	#/rel Saving	5 9%	0.80 11%	8 14%	0.85 11%	5 14%	0.90 11%	8 19%	0.95 19%	5 14%	0.90 14%	8 19%	0.95 19%
Cognitive functioning	#/rel Saving	3 20%	0.80 22%	5 27%	0.90 27%	4 27%	0.90 27%	6 28%	0.95 28%	4 25%	0.90 23%	6 28%	0.95 28%
Social functioning	#/rel Saving	3 12%	0.75 14%	5 19%	0.85 17%	4 19%	0.90 19%	6 27%	0.95 27%	4 27%	0.90 23%	6 30%	0.95 30%
Fatigue	#/rel Saving	4 16%	0.85 16%	6 19%	0.95 19%	5 14%	0.90 11%	7 19%	0.95 16%	5 16%	0.90 14%	8 19%	0.95 19%
Nausea and vomiting	#/rel Saving	4 42%	0.85 42%	7 45%	0.90 42%	4 48%	0.90 48%	7 52%	0.95 52%	4 48%	0.90 48%	7 50%	0.95 48%
Pain	#/rel Saving	3 3%	0.80 17%	6 19%	0.85 19%	4 19%	0.90 19%	7 23%	0.95 25%	4 19%	0.90 19%	7 25%	0.95 25%
Dyspnoea	#/rel Saving	3 36%	0.80 38%	5 39%	0.85 38%	4 41%	0.90 34%	6 44%	0.95 39%	4 34%	0.90 28%	6 38%	0.95 37%
Insomnia	#/rel Saving	3 27%	0.75 25%	5 31%	0.90 31%	4 30%	0.90 27%	6 34%	0.95 36%	4 28%	0.90 28%	6 33%	0.95 34%
Lack of appetite	#/rel Saving	3 22%	0.80 22%	5 27%	0.90 27%	3 23%	0.90 22%	5 31%	0.95 30%	4 34%	0.90 27%	6 38%	0.95 34%
Constipation	#/rel Saving	3 27%	0.80 27%	6 34%	0.90 31%	3 31%	0.90 28%	6 39%	0.95 38%	3 38%	0.90 34%	6 42%	0.95 39%
Diarrhoea	#/rel Saving	3 17%	0.85 19%	6 22%	0.90 20%	4 31%	0.90 25%	6 34%	0.95 31%	3 34%	0.90 30%	5 38%	0.95 34%
Financial difficulties	#/rel Saving	3 27%	0.65 23%	5 28%	0.90 27%	3 27%	0.90 23%	5 34%	0.95 31%	3 30%	0.90 27%	5 36%	0.95 33%
Median across 14 domains	#/rel Saving	3 20%	0.80 21%	6 25%	0.90 25%	4 24%	0.90 21%	6 30%	0.95 28%	4 27%	0.90 23%	6 31%	0.95 29%

Requiring the CATs to start with a QLQ-C30 item and to include items from all subdomains generally seemed to have only trivial impact on precision and power except in a few cases for very short CATs asking < 4 items and did not seem to reduce measurement performance of the suggested standard settings. The impact of stopping if the first items were answered ‘not at all’ varied across domains and populations, but generally seemed a viable option although it typically reduced precision and power slightly.

## Discussion

IRT calibrated item banks have the major advantage that any subset of items from a bank provides directly comparable scores. When basing a PROM on an item bank, this property offers great flexibility for optimising the PROM to the requirements of a study without compromising comparability of scores. CAT-assessments further utilise this to select the most relevant items (in terms of targeting) to the individual, generally resulting in more efficient and precise measurement [5]. However, the advantages of CAT-assessment depend crucially on how the CAT is set up to run, i.e. the CAT-setting that is used for the assessment. Selecting the ‘optimal’ CAT-setting may not be simple. To simplify this selection when using the EORTC CAT Core, we evaluated a collection of CAT-settings, identifying the optimal settings for measurement in one of three populations: patients typically having mild, moderate, or severe symptoms, respectively. We use the term ‘standard CAT-settings’ to indicate that they specify predefined CAT designs with predicted performances in certain populations. Standard does not indicate these are the standard for how CAT-assessments should be conducted with the EORTC CAT Core. Clearly, not all possible CAT-settings can be evaluated. Hence, there may be settings not evaluated here having more desirable properties for specific situations. Rather, the standard settings should be viewed as suggestions with ‘known’ properties that may be used as they are or serve as a sensible starting point for designing a customised CAT-setting fulfilling the specific requirements of a study. Particularly, in situations deviating from the scenarios evaluated here there may be a need for customisation. In such cases, the suggested settings may still provide indications on the basic design of the ‘optimal’ CAT-setting.

Although these standard CAT-settings aim to simplify the choice of setting it may still be a complex process—what is the level of symptomatology of the target population, should it be fixed-length, fixed-precision, long, brief etc. If limited or no information is available about symptomatology, then a small pilot study may be considered to obtain initial estimates. For researchers not familiar with CAT it may not be simple to choose between the different CAT options. However, selecting an EORTC CAT-setting is usually done in close collaboration with the EORTC Quality of Life Department. This ensures the researcher receive sufficient knowledge about the different settings to make informed decisions.

We focused on the two basic types of CATs, fixed-length and fixed-precision. But the optimal setting for a given situation may be a mix of these, i.e. the CAT stops after a specified number of items or when a specified precision has been obtained, whatever comes first. Additional selection and/or stopping criteria may also be needed. We evaluated one such additional stopping criterion, namely stopping if the first items were answered ‘not at all’ regardless of whether the basic stop criterion (length/precision) was fulfilled. This was examined since asking several questions to patients not having the symptom of focus provides only trivial additional information (e.g. asking many items on constipation to a patient not being constipated provides limited information). Patients without a symptom may also find it particularly needless or even annoying to be asked several questions about the symptom [18]. Adding this criterion obviously reduced the number of items asked for those having low levels of a symptom but typically also reduced measurement precision slightly. Hence, when efficiency, i.e. low response time and burden, is a priority and/or when high measurement precision among patients with very low levels of symptoms is not important (e.g. when aiming to identify patients with symptoms above a given threshold likely to require treatment [19]) adding this stopping criterion may be desirable. When measuring several domains reducing the number of ‘unnecessary’ items may be particularly relevant to keep the total number of items asked at an acceptable level.

When selecting the standard CAT-settings we gave priority to both measurement precision and content. Content was taken into consideration by initiating the CATs with an QLQ-C30 item, ensuring a direct link to the original instrument, and by requiring that items from all subdomains of a domain were included, thereby covering the full content of a domain in all assessments. Although content-based restrictions in general may reduce the measurement precision of CATs, our evaluations indicated that the content requirement examined here had only trivial impact on measurement performance. If content is not considered, CAT-assessments may include items from some subdomains only. For example, when assessing physical functioning some patients might only be asked items on walking/moving while others were only asked items on lifting/carrying objects. Such ‘narrow’ assessments are typically undesirable as they may introduce interindividual biases (e.g. some patients might not be able to walk over long distances but may be capable of carrying heavy objects). Hence, it is typically advisable to take content into account but at the same time ensure that such content considerations do not significantly impact measurement properties. For the suggested standard CAT-settings, a sensible balance between content considerations and measurement precision was reached.

To assist in judging the practical impact of choosing a CAT-setting, we simulated the expected relative sample size savings of using the CATs compared to using the QLQ-C30 scales. Considerable variation was observed across the 14 domains. Several factors may contribute to these variations, some of the most important likely being the number of items in the bank informative for the target population and the level of information of the C30 scale for the population. How these and other factors interact and affect findings is complex and may be an area for further research. In any case, all suggested CAT-settings resulted in higher power and hence, lower sample size requirements than the QLQ-C30 scales.

Although we assessed various group differences and group sizes, the simulations were clearly not exhaustive. Hence, the findings may not generalise to all other settings, e.g. for assessing changes over time or differences in populations deviating markedly from the populations investigated here. Clearly, many alternative approaches for designing CAT-settings exist which may also result in viable settings. Further, note that the provided estimated savings are averages (medians) and variation across individual studies should be expected. Still, the estimated savings may provide valuable indication of the performance of a CAT and the practical impact of choosing one CAT-setting over another.

## Conclusion

Applying a general approach for identifying ‘optimal’ CAT-settings which balances efficiency, precision, and content, a collection of standard settings for the 14 domains of the EORTC CAT Core was obtained. The settings were optimised for assessment in populations with predominantly mild, moderate, and severe symptoms, respectively. Simulations indicated that the CATs generally provide increased power and reduced sample size requirements as compared to the QLQ-C30. Average sample savings using the CATs were approximately 20–30%, although considerable variation across domains were observed. The collection of standard CAT-settings allows for simple selection of settings with ‘known’ measurement properties when using the EORTC CAT Core. The suggested settings may be used as they are or as starting points for designing customised CAT-settings fulfilling the specific requirements of a study. Further information regarding the use of the EORTC CAT Core may be obtained at https://qol.eortc.org/cat/.

### Supplementary Information

Below is the link to the electronic supplementary material.

Supplementary file1 (DOCX 1942 KB)
