# Supplementary material for: Development of standard computerised adaptive test (CAT) settings for the EORTC CAT Core
Source: Qual Life Res. 2024 Jan 17;33(4):951–61. doi: 10.1007/s11136-023-03576-x (PMC10972921; doi:10.1007/s11136-023-03576-x)
Supplement: Supplementary file 1 — Supplementary file1 (DOCX 1942 KB) [file 11136_2023_3576_MOESM1_ESM.docx]

Supplemental material

Article: Development of standard CAT (computerised adaptive test) settings for the EORTC CAT Core

Journal: Quality of life Research

Authors: Morten Aa. Petersen^*^, Hugo Vachon, Johannes M. Giesinger & Mogens Groenvold on behalf of the European Organisation for Research and Treatment of Cancer (EORTC) Quality of Life Group

* Corresponding author: Palliative Care Research Unit, Department of Geriatrics and Palliative Medicine GP, Bispebjerg & Frederiksberg Hospital, Bispebjerg bakke 23B, 2400 Copenhagen NV, Denmark. Telephone: (+45) 3863 5016. Fax: (+45) 3863 9805. Email: [Morten.Aagaard.Petersen@regionh.dk](mailto:Morten.Aagaard.Petersen@regionh.dk). ORCID: 0000-0002-3117-632X

This supplemental material provides three things:

1) A table of all start items for the standard CAT-settings. For each target population (mild, moderate, or severe) brief and long versions apply the same start item. All the standard CATs apply maximum Fisher information for item selection and EAP for score estimation and all fixed-precision CATs ask a maximum of eight items. Hence, this table along with Table 3 of the article provides all information needed to design the standard CAT-settings.

2) The simulated relative validities and relative sample sizes of using the EORTC CAT Core compared to using the QLQ-C30 for all 14 item banks of the EORTC CAT Core. For each item bank this first shows the relative validities and relative sample sizes for different fixed-length CATs for the three target populations, i.e., patients having predominantly mild, moderate, and severe symptoms/problems, respectively (in that order) followed by the relative validities and relative sample sizes for different fixed-precision CATs (same order of target populations).

3) SAS code for conducting the CAT simulations used for evaluating the measurement properties of different CAT-settings. The code does not include the actual item parameters as these are intellectual property of the EORTC Quality of Life Group. The code is configured for simulations of CATs for the physical functioning item bank. The base of the code is applicable to simulations of any of the item banks but needs adjustment to the number of items of the specific item bank.

# Start items for the standard CAT-settings for the three target populations

| Domain/population | Mild | Moderate | Severe |
| --- | --- | --- | --- |
| Physical functioning | Do you have any trouble taking a long walk? | Do you have any trouble taking a short walk outside of the house? | Do you have any trouble taking a short walk outside of the house? |
| Role functioning | Were you limited in doing either your work or other daily activities? | Were you limited in doing either your work or other daily activities? | Were you limited in doing either your work or other daily activities? |
| Emotional functioning | Did you feel depressed? | Did you feel depressed? | Did you feel depressed? |
| Cognitive functioning | Have you had difficulty remembering things? | Have you had difficulty remembering things? | Have you had difficulty remembering things? |
| Social functioning | Has your physical condition or medical treatment interfered with your social activities? | Has your physical condition or medical treatment interfered with your social activities? | Has your physical condition or medical treatment interfered with your family life? |
| Fatigue | Were you tired? | Were you tired? | Have you felt weak? |
| Nausea & vomiting | Have you felt nauseated? | Have you felt nauseated? | Have you felt nauseated? |
| Pain | Did pain interfere with your daily activities? | Did pain interfere with your daily activities? | Did pain interfere with your daily activities? |
| Dyspnoea | Were you short of breath? | Were you short of breath? | Were you short of breath? |
| Insomnia | Have you had trouble sleeping? | Have you had trouble sleeping? | Have you had trouble sleeping? |
| Lack of appetite | Have you lacked appetite? | Have you lacked appetite? | Have you lacked appetite? |
| Constipation | Have you been constipated? | Have you been constipated? | Have you been constipated? |
| Diarrhoea | Have you had diarrhoea? | Have you had diarrhoea? | Have you had diarrhoea? |
| Financial difficulties | Has your physical condition or medical treatment caused you financial difficulties? | Has your physical condition or medical treatment caused you financial difficulties? | Has your physical condition or medical treatment caused you financial difficulties? |

# Physical functioning, fixed-length CATs


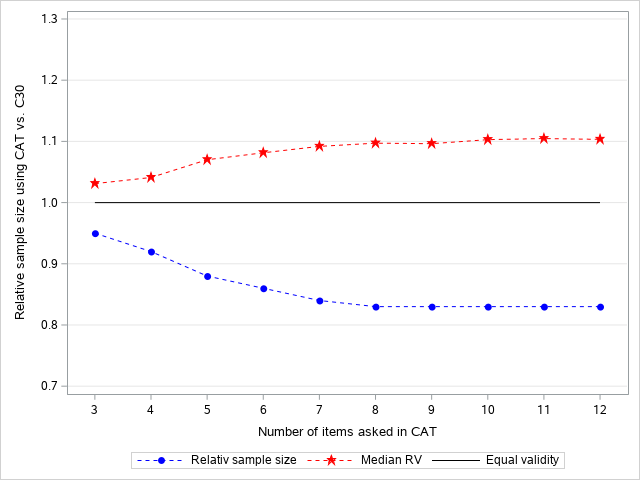

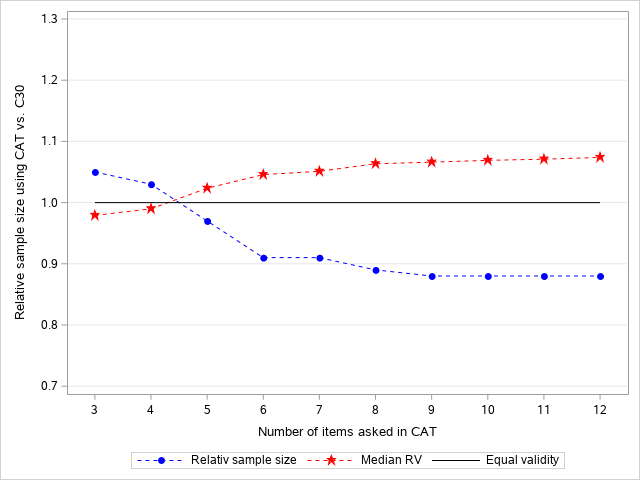

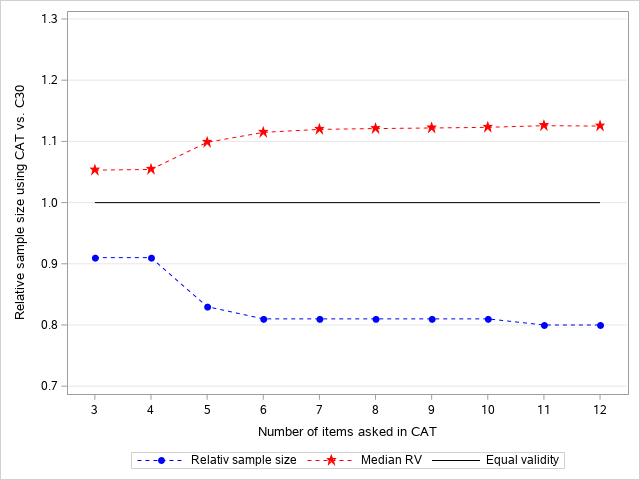


# Physical functioning, fixed-precision CATs


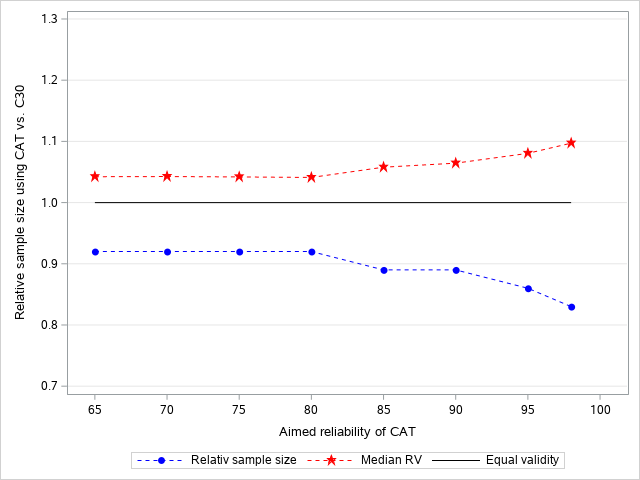


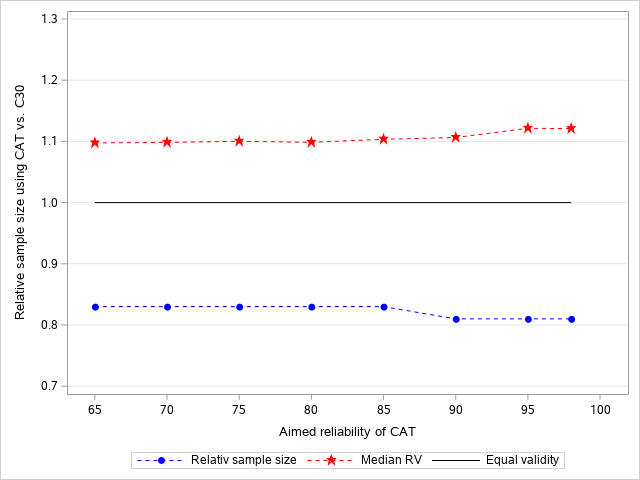

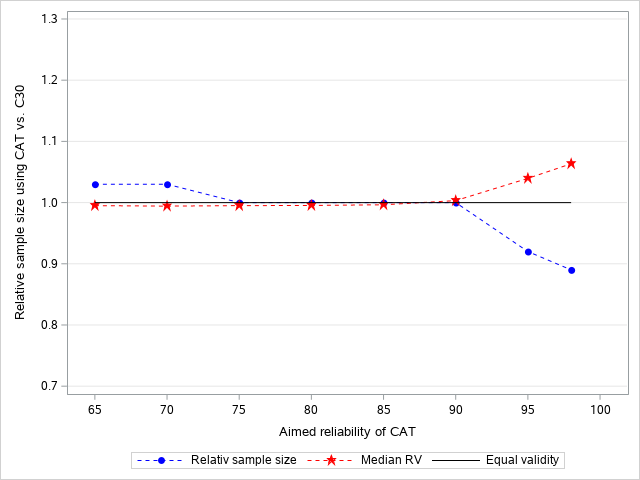


# Role functioning, fixed-length CATs


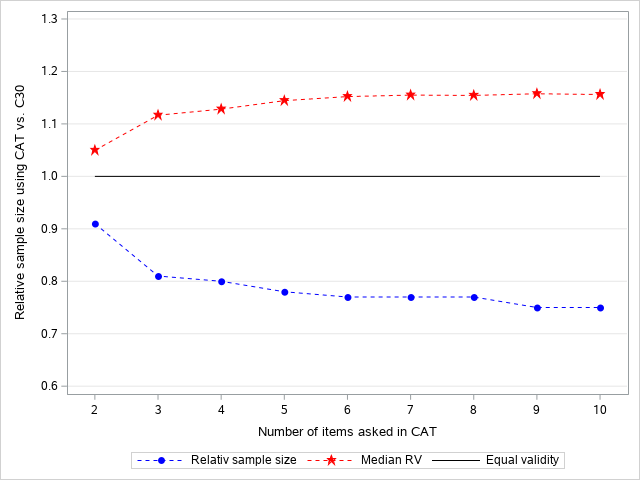


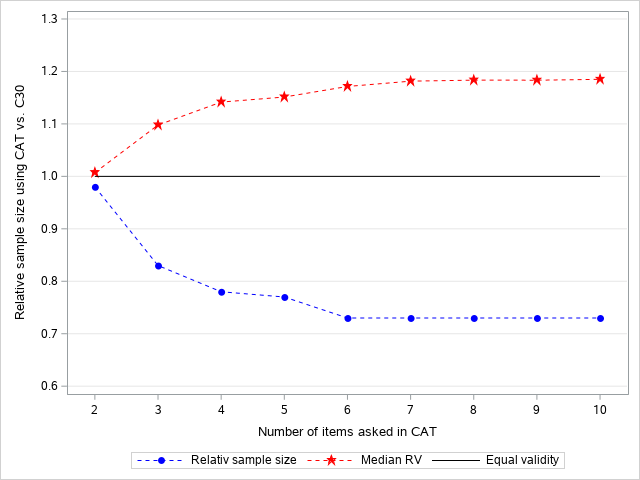


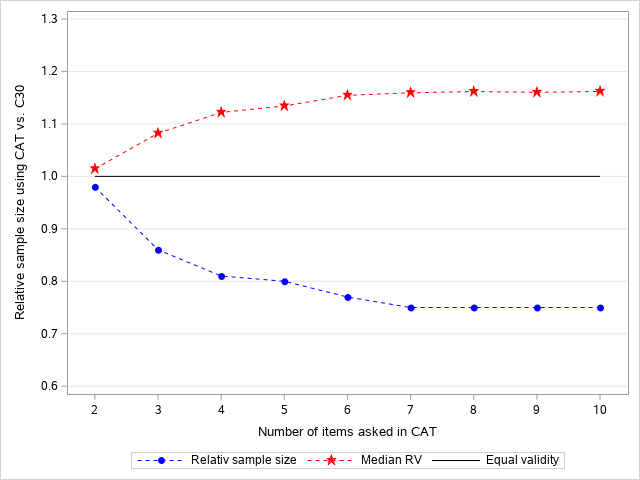


# Role functioning, fixed-precision CATs


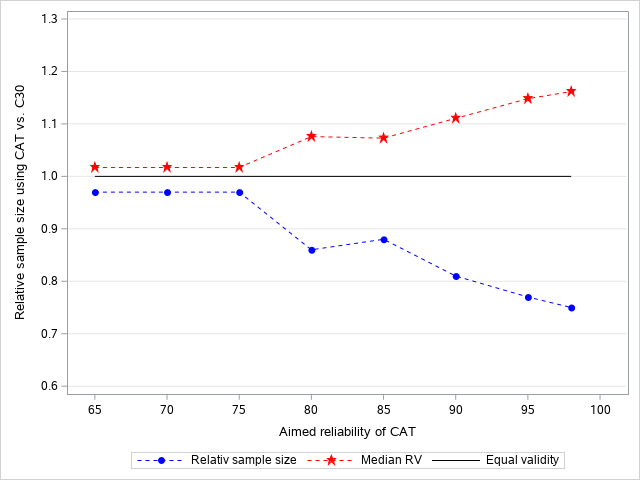


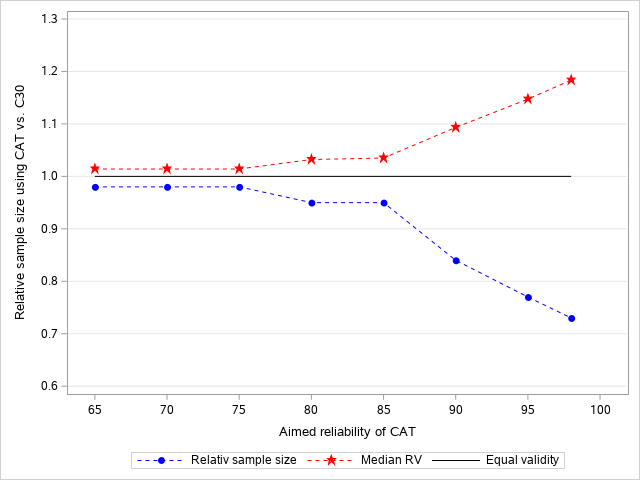

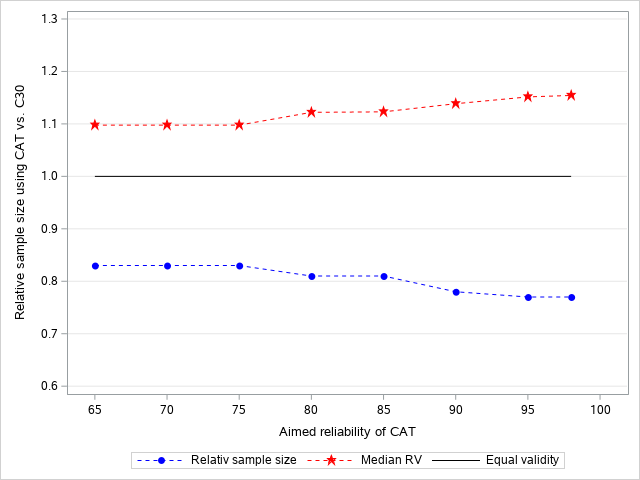


# Emotional functioning, fixed-length CATs


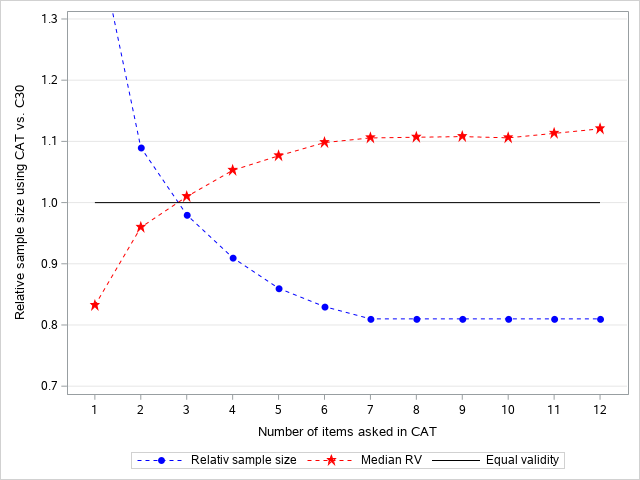


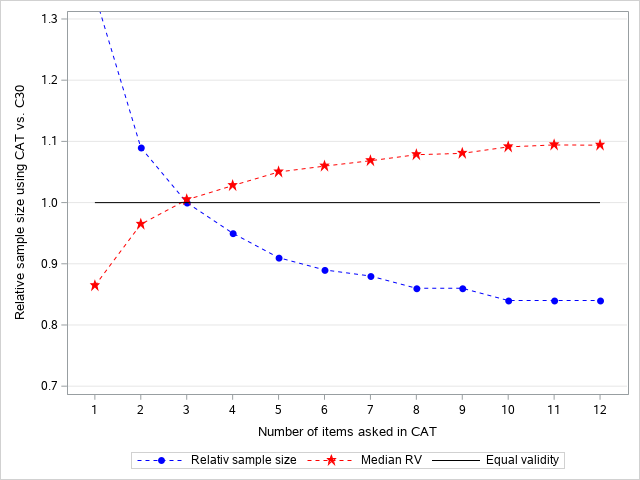

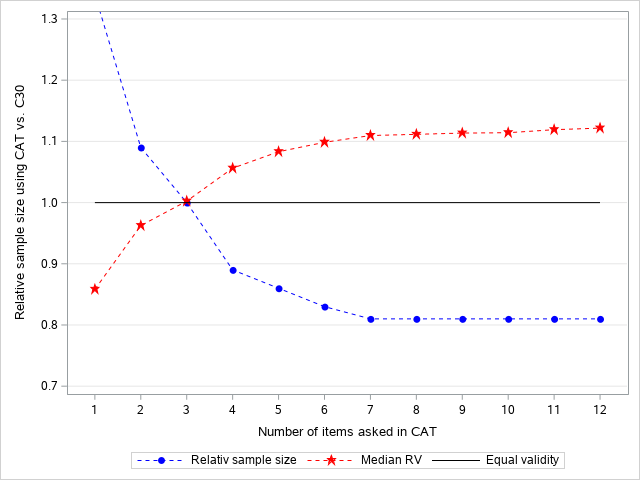


# Emotional functioning, fixed-precision CATs


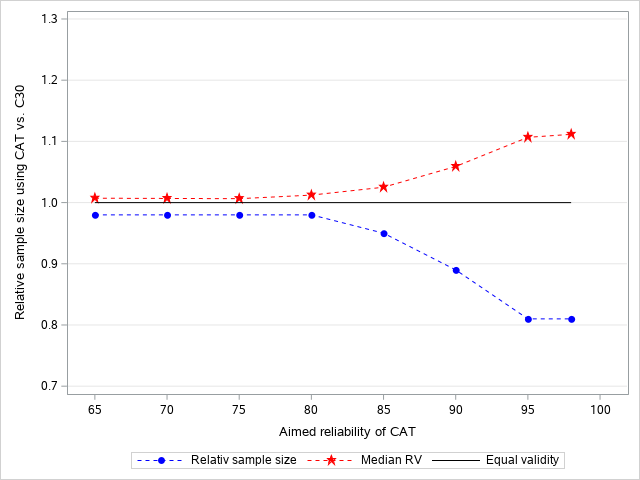

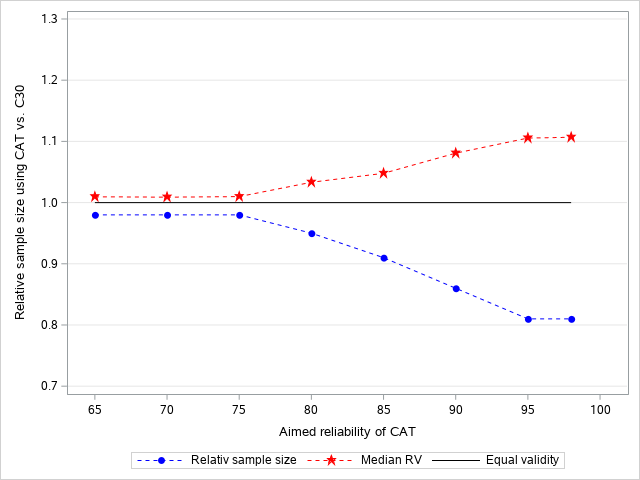

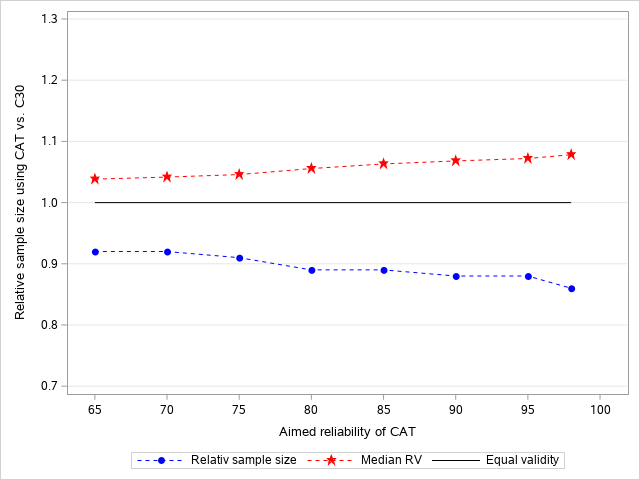


# Cognitive functioning, fixed-length CATs


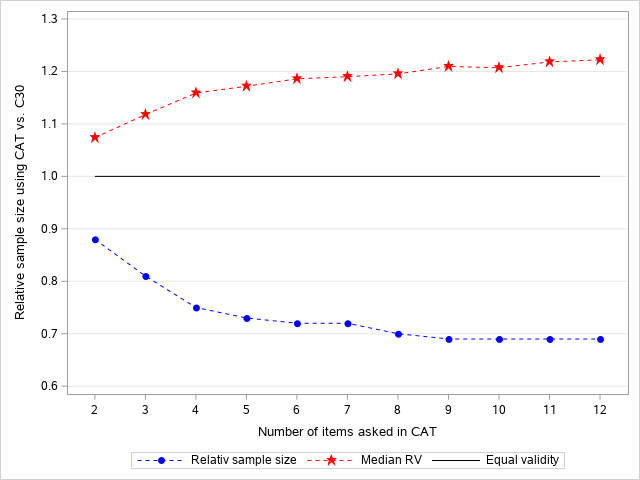


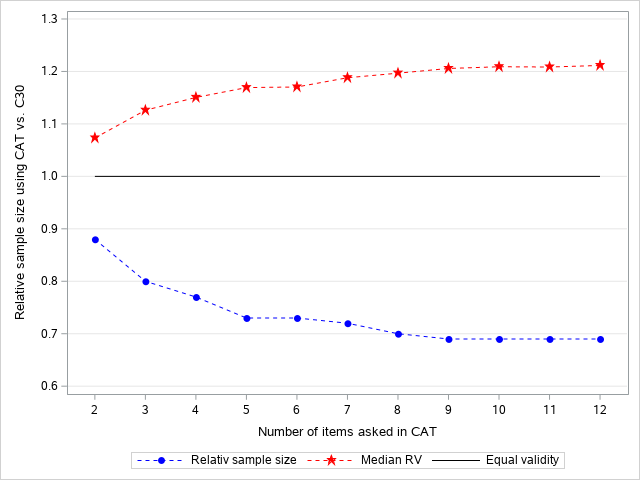

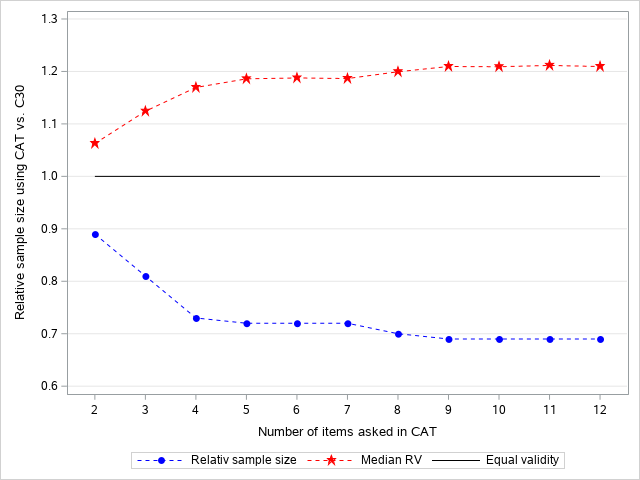


# Cognitive functioning, fixed-precision CATs


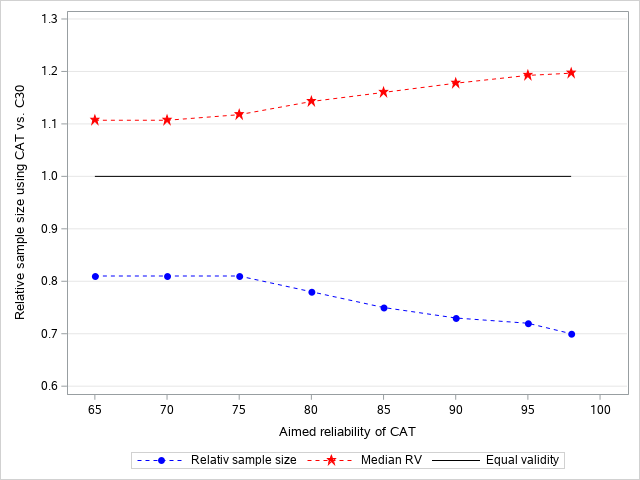


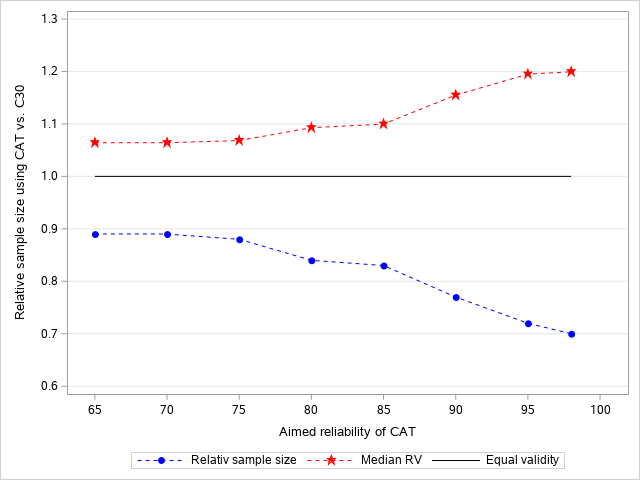

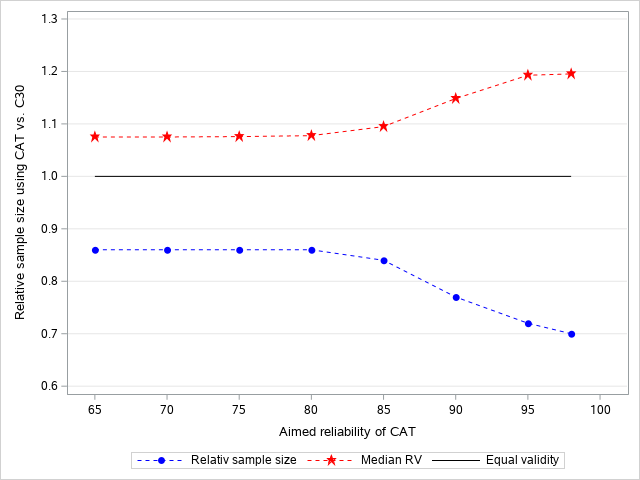


# Social functioning, fixed-length CATs


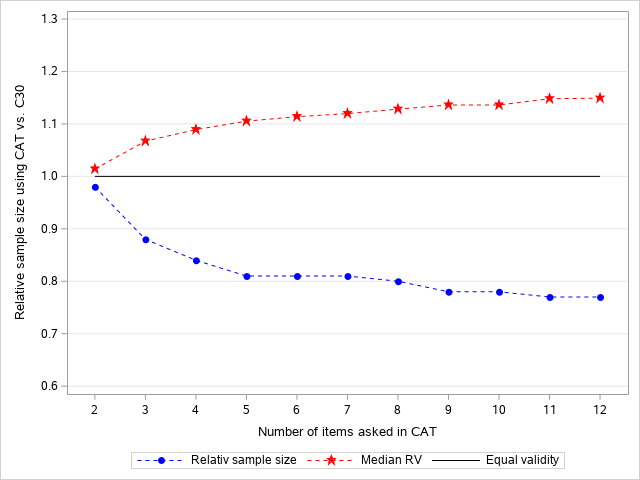


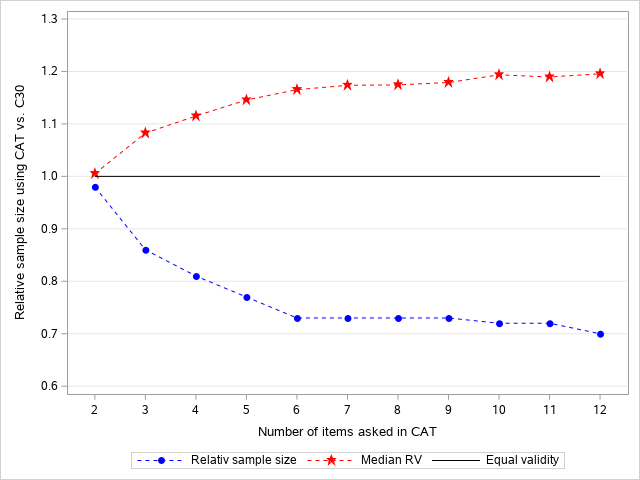

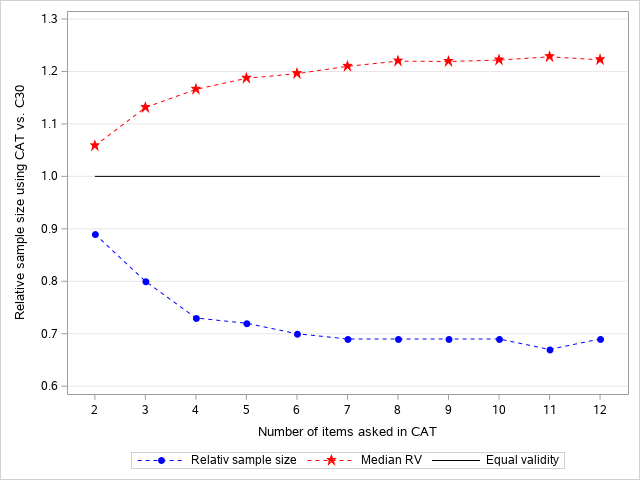


# Social functioning, fixed-precision CATs


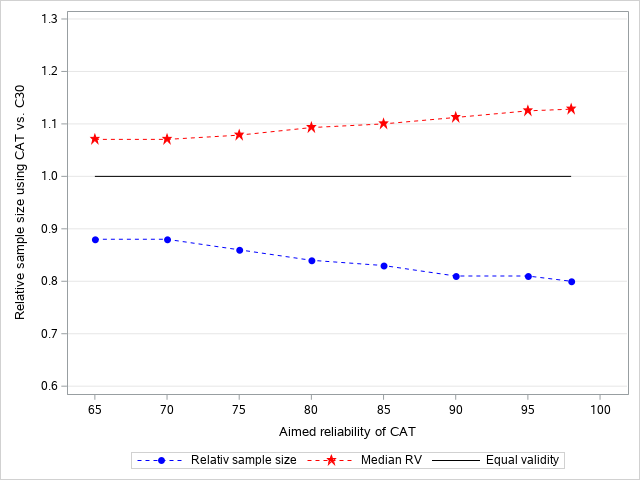


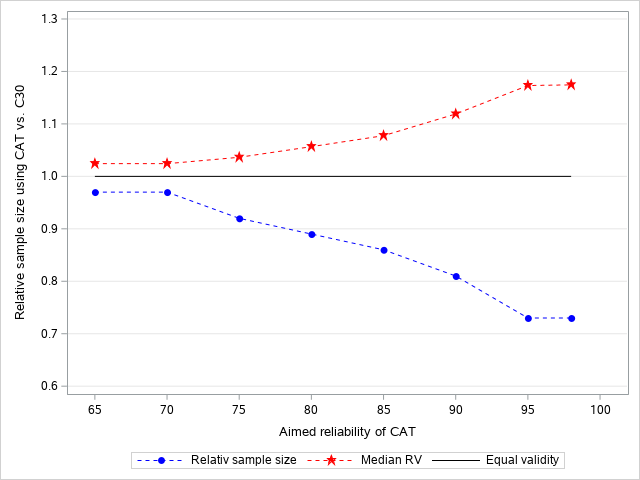

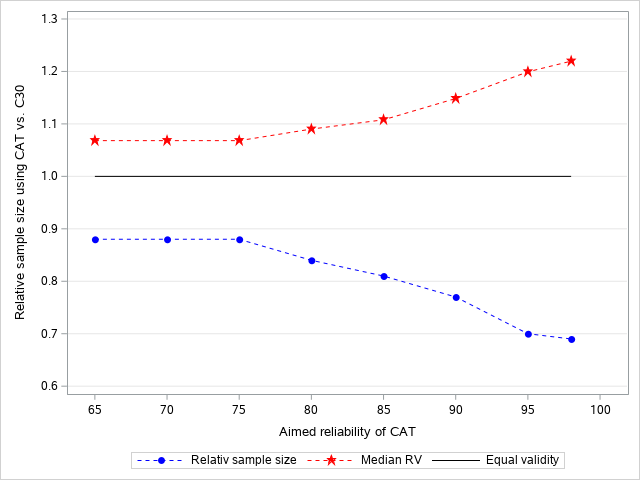


# Fatigue, fixed-length CATs


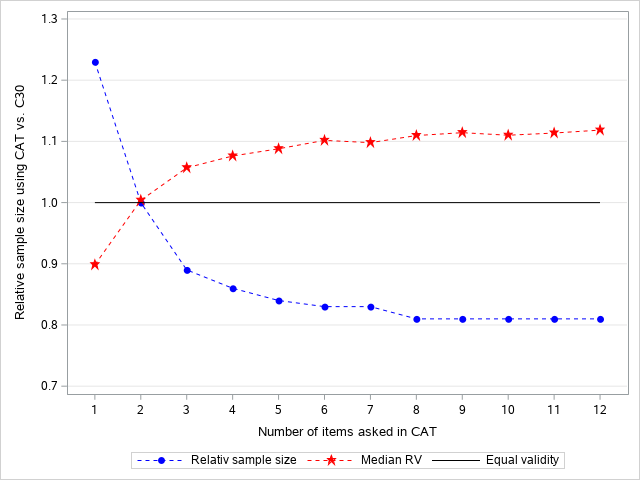


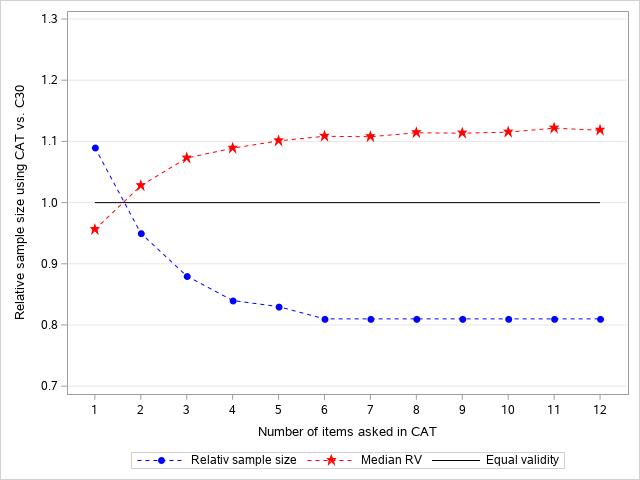

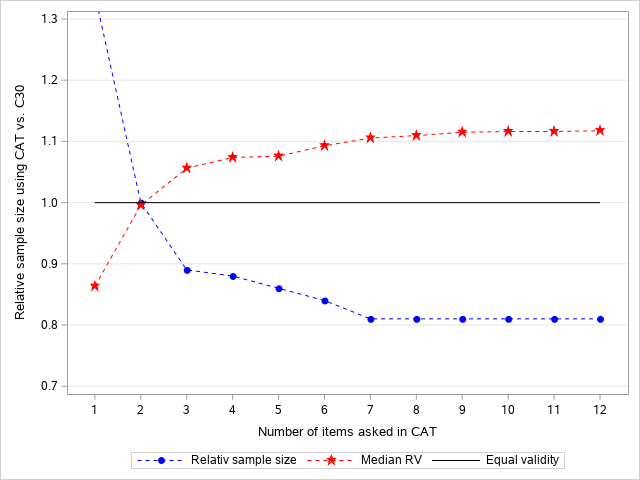


# Fatigue, fixed-precision CATs


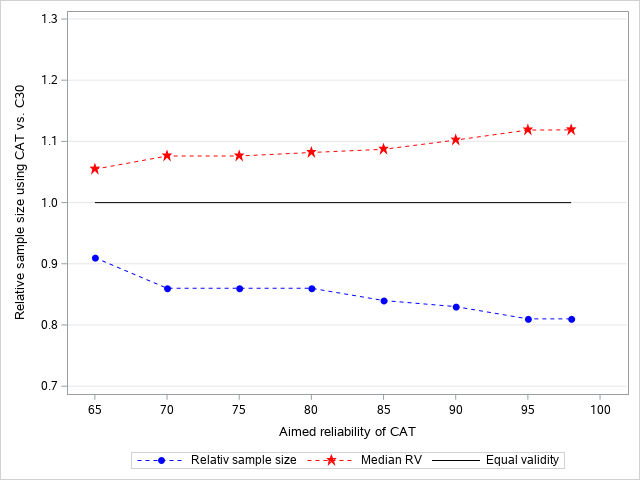


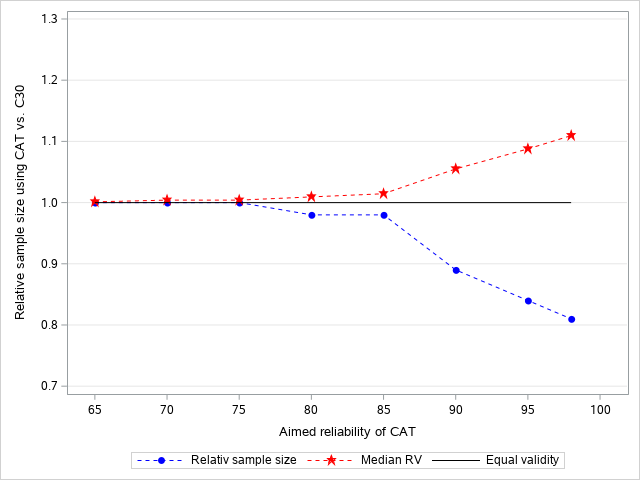

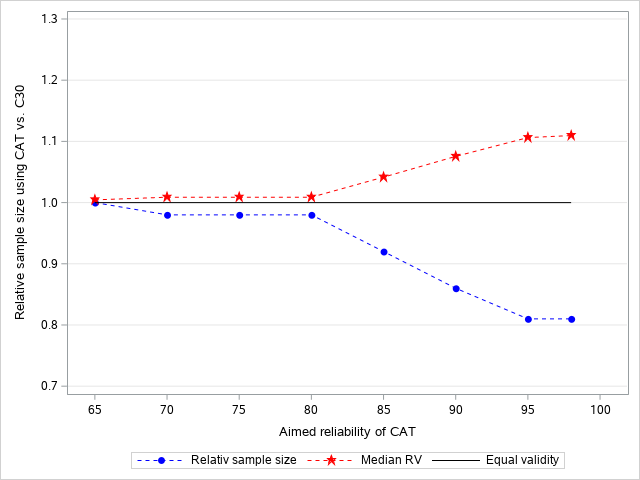


# Nausea & vomiting, fixed-length CATs


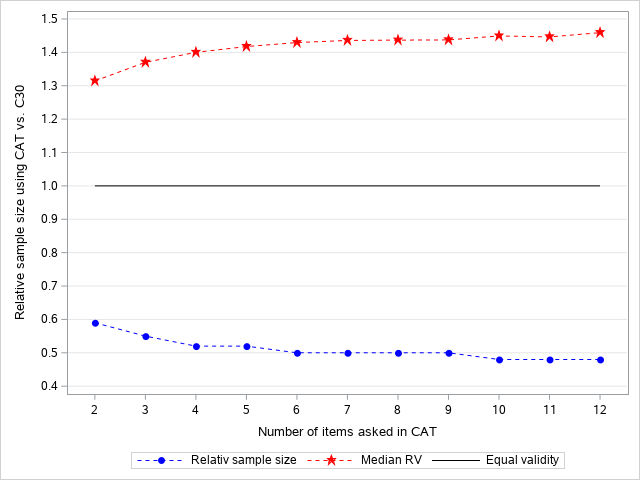


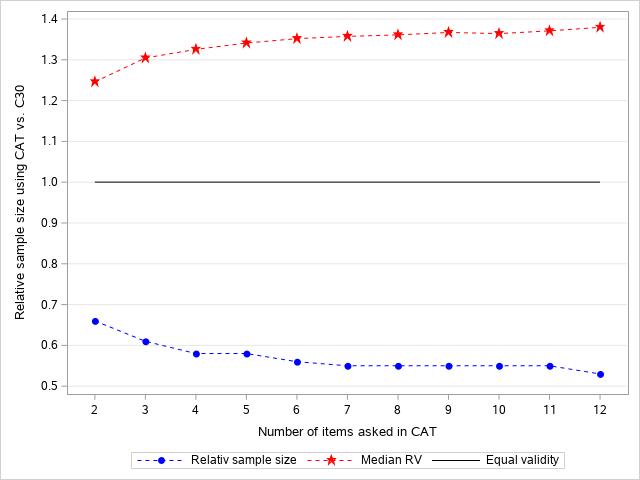

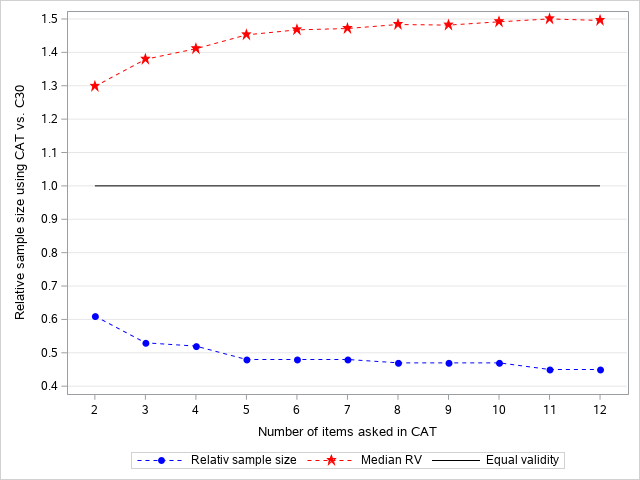


# Nausea & vomiting, fixed-precision CATs


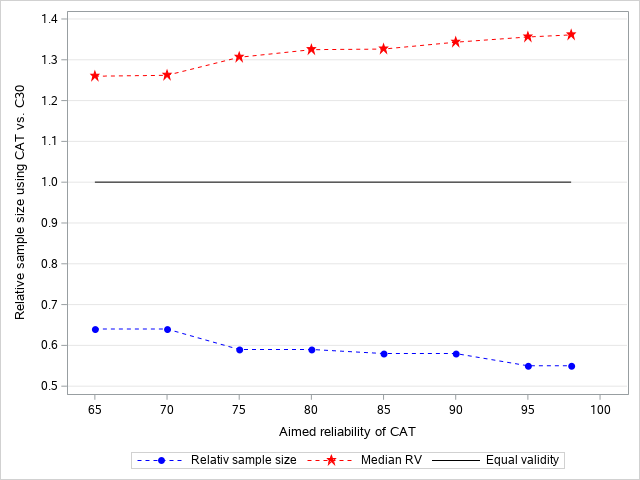


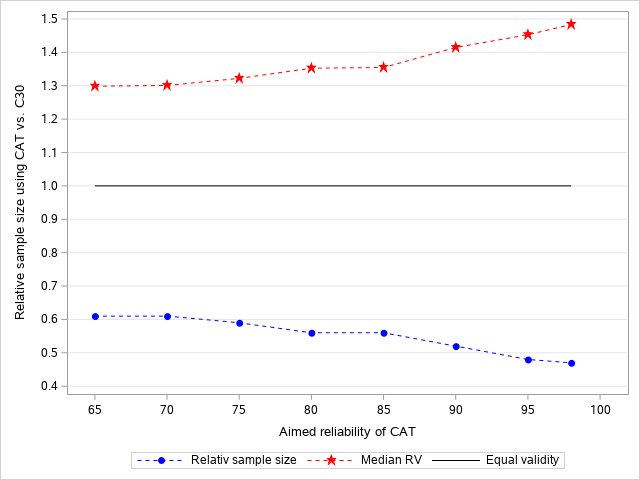

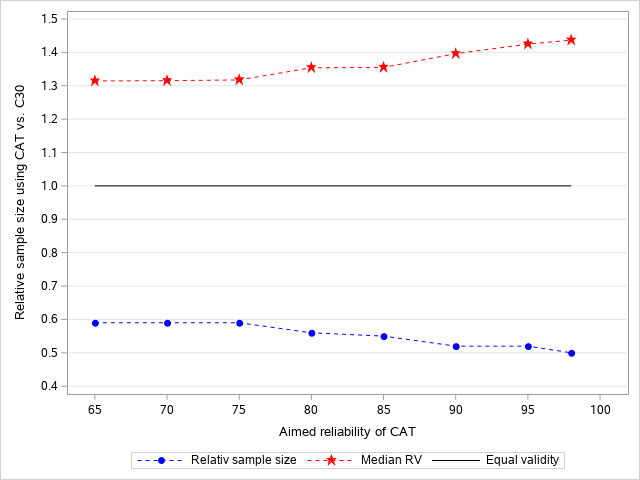


# Pain, fixed-length CATs


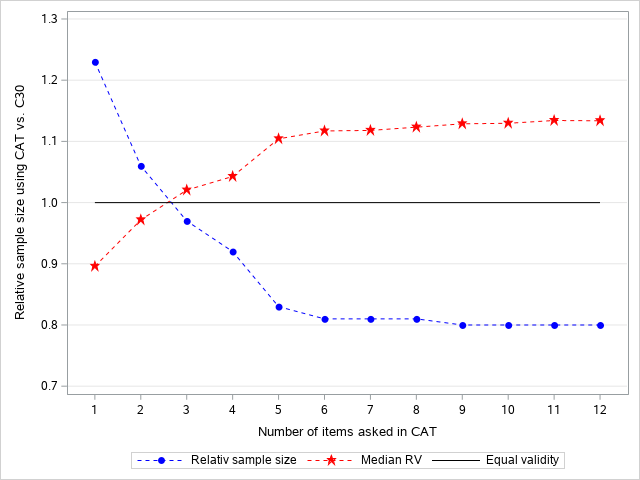


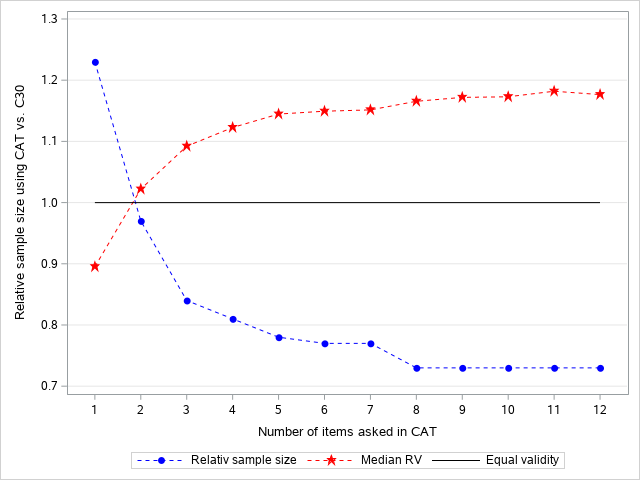

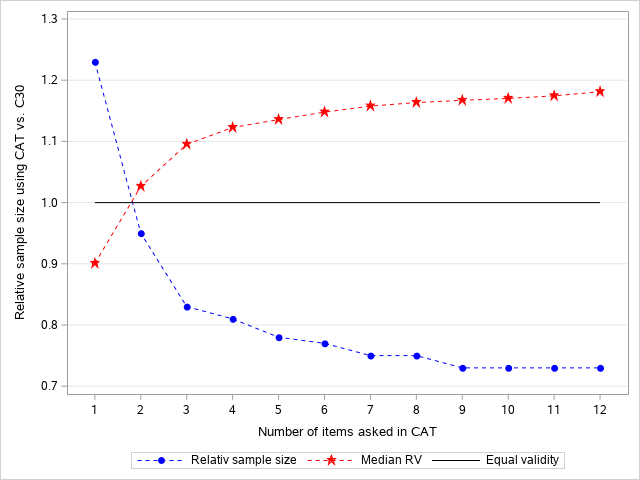


# Pain, fixed-precision CATs


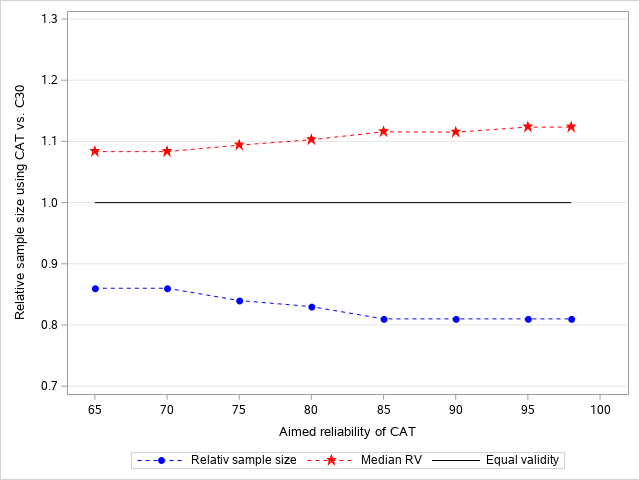


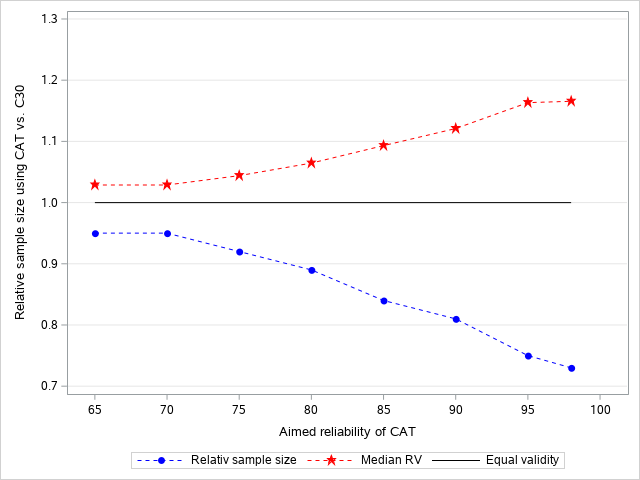

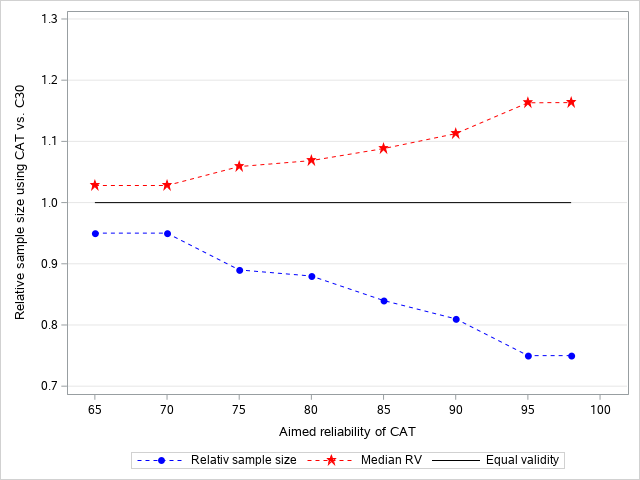


# Dyspnoea, fixed-length CATs


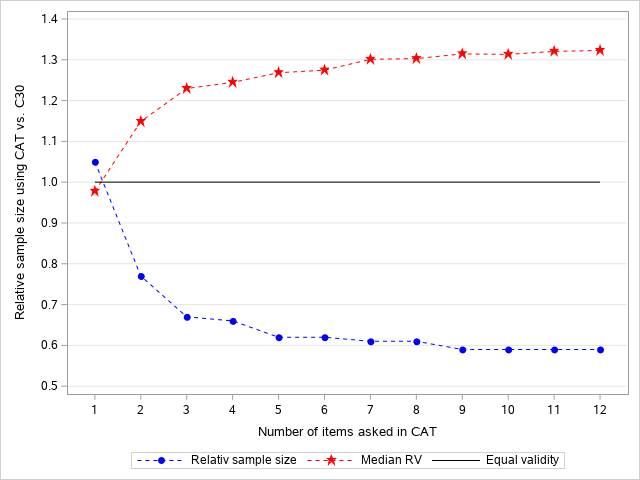


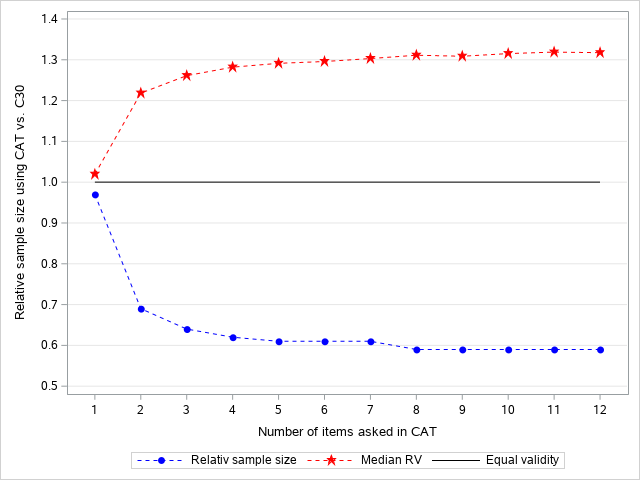

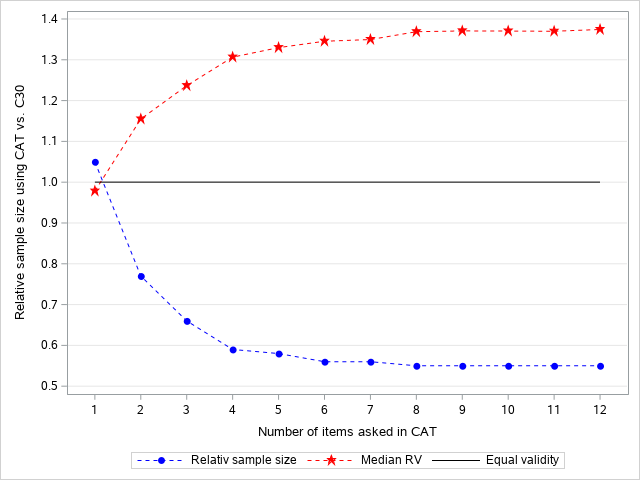


# Dyspnoea, fixed-precision CATs


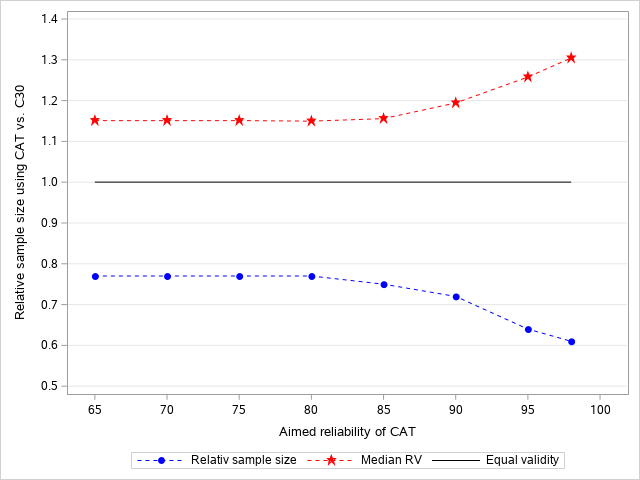


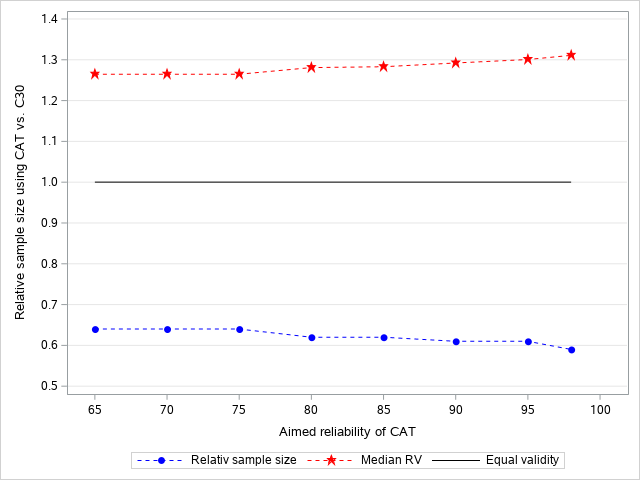

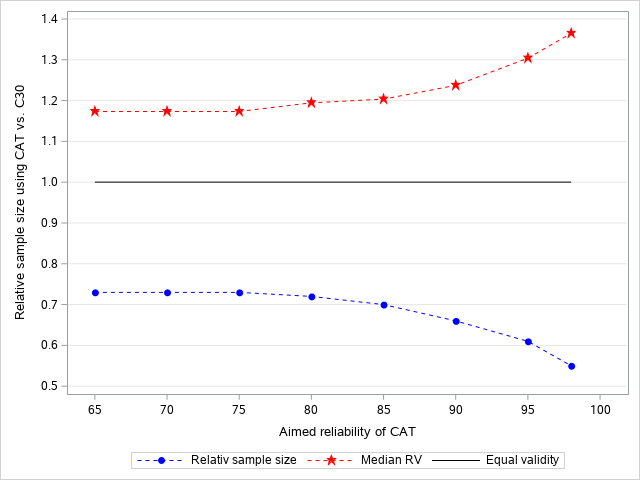


# Insomnia, fixed-length CATs


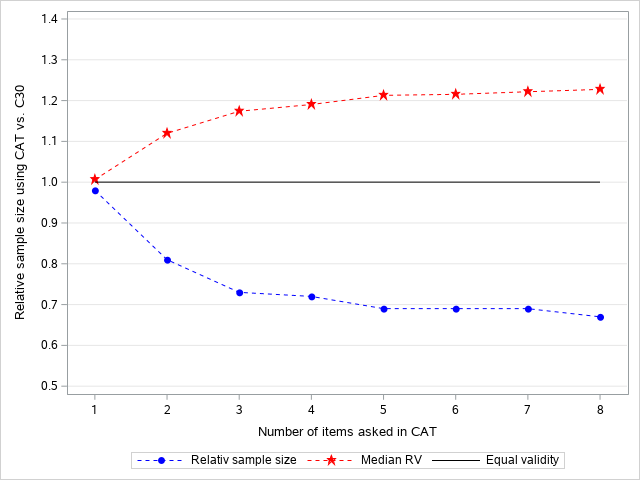


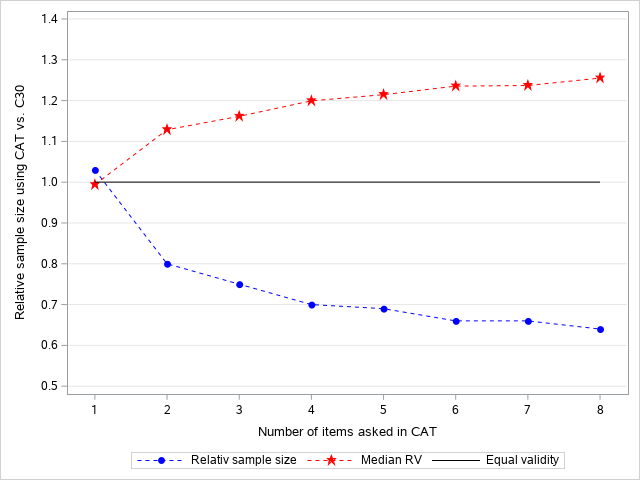

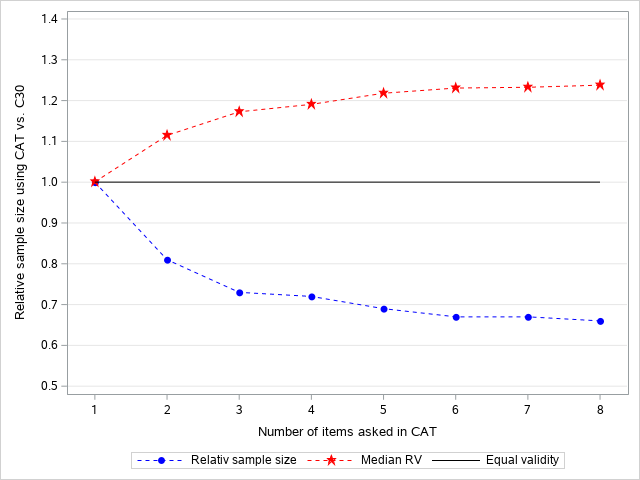


# Insomnia, fixed-precision CATs


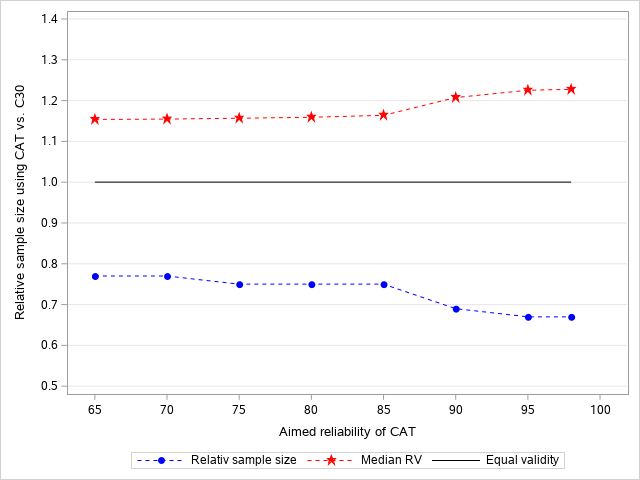


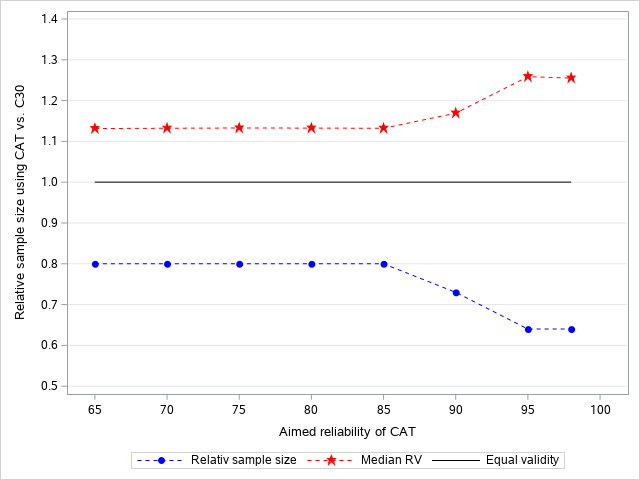


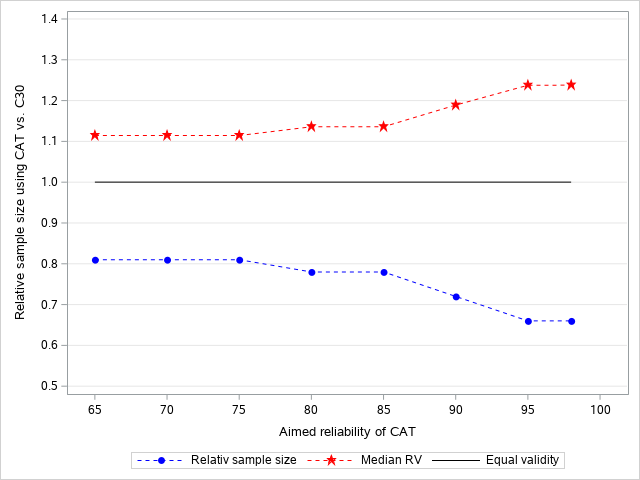


# Lack of appetite, fixed-length CATs


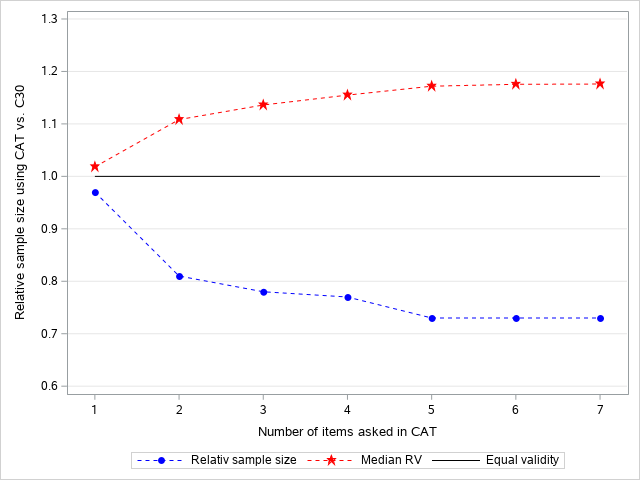


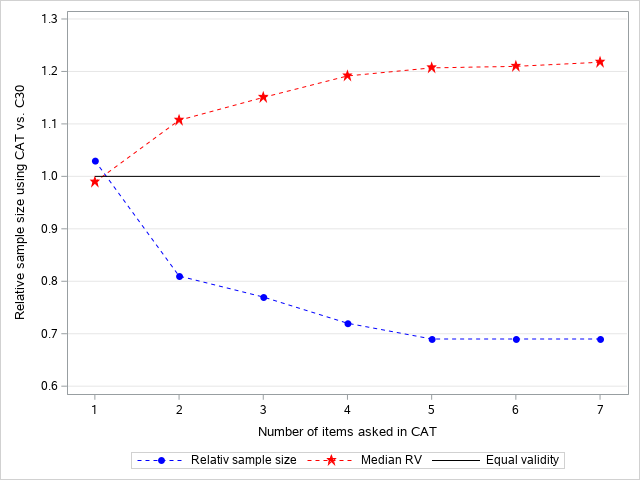

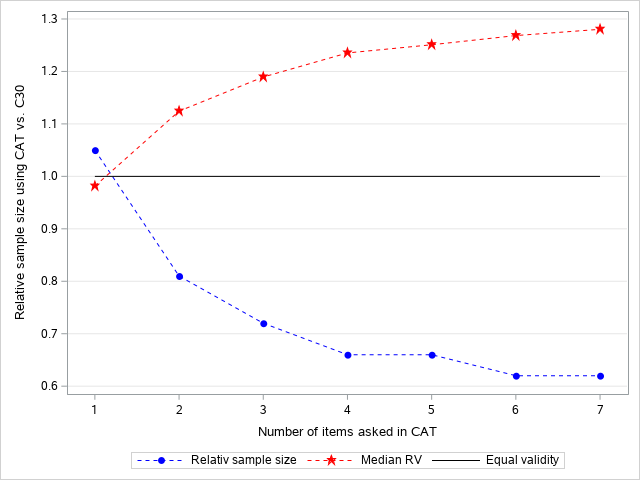


# Lack of appetite, fixed-precision CATs


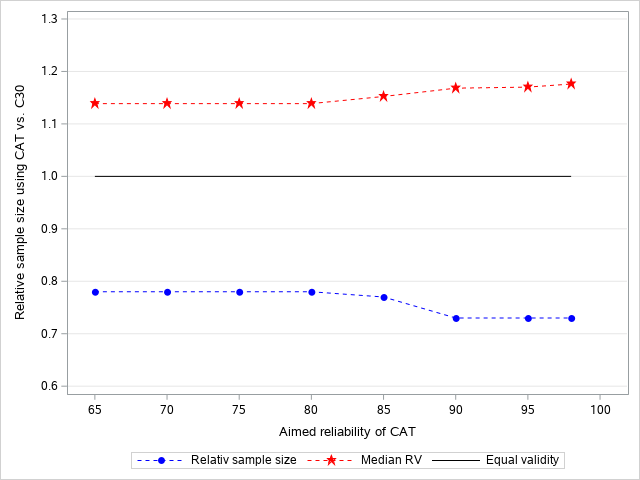


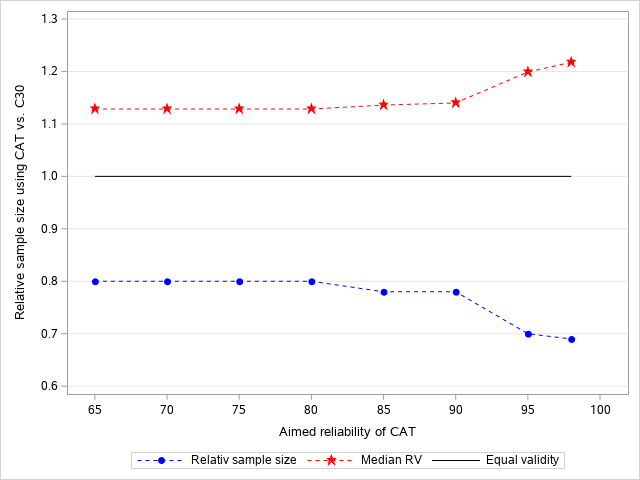


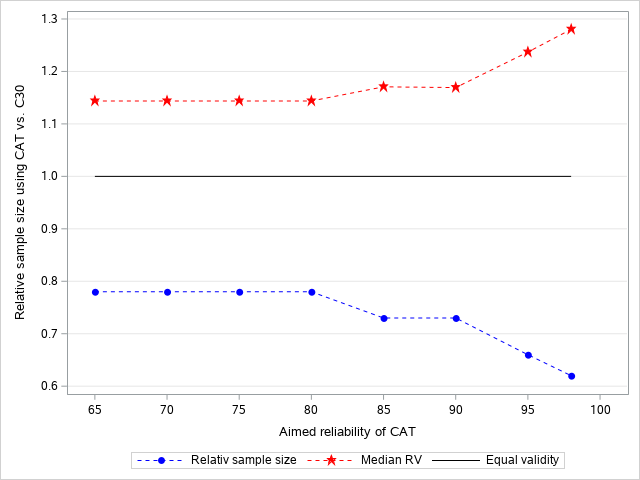


# Constipation, fixed-length CATs


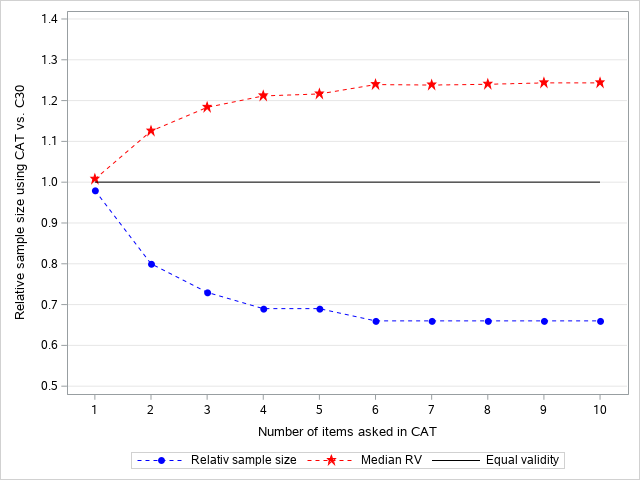


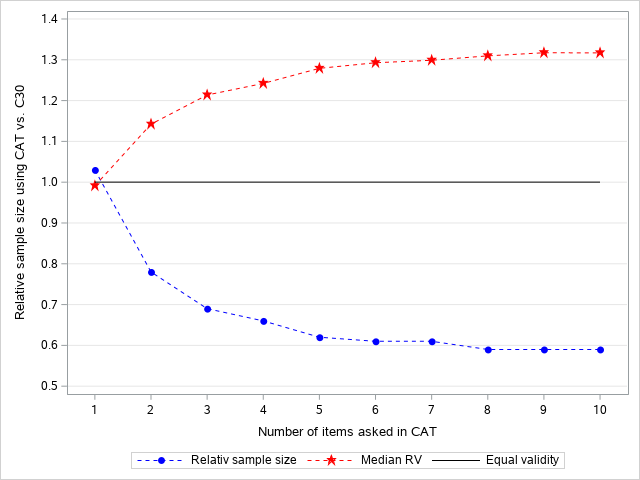

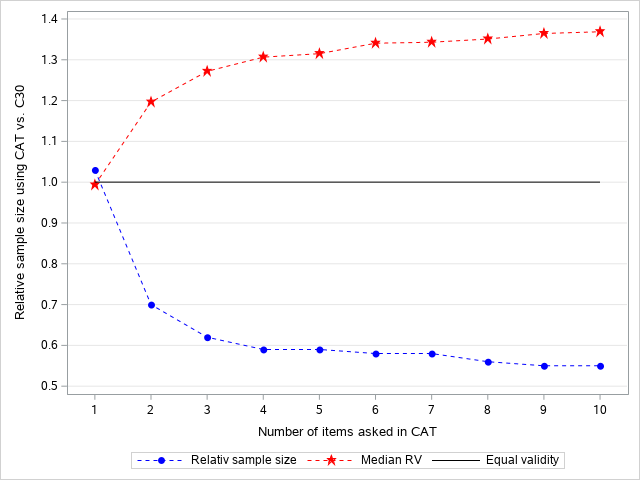


# Constipation, fixed-precision CATs


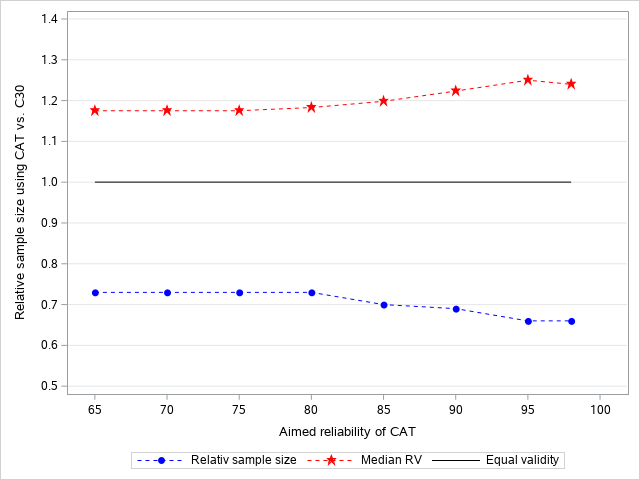


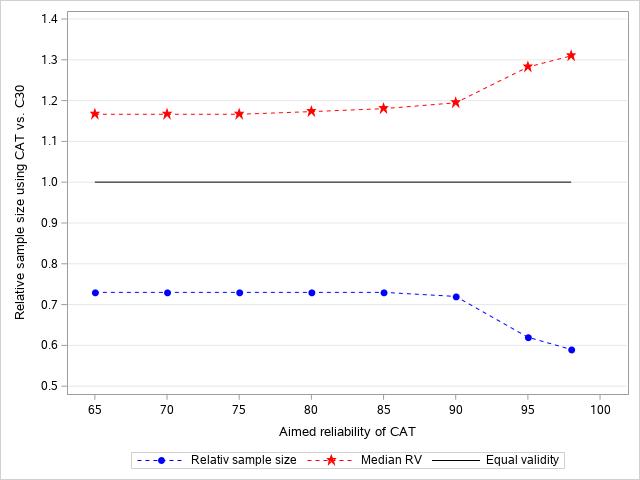

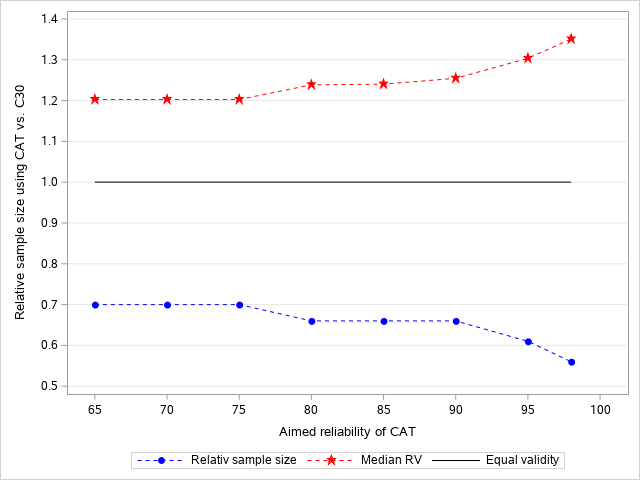


# Diarrhoea, fixed-length CATs


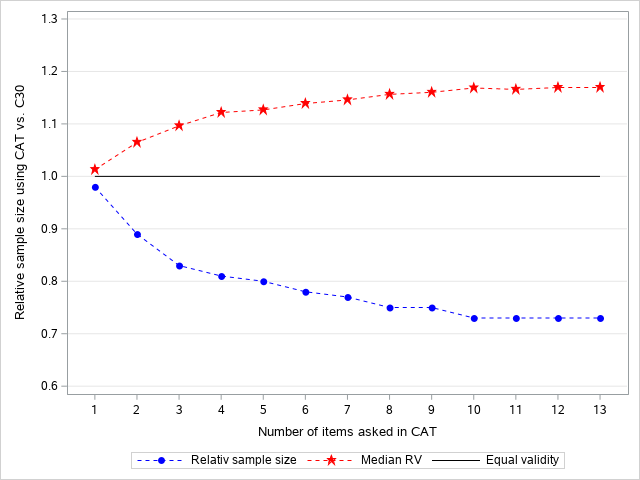


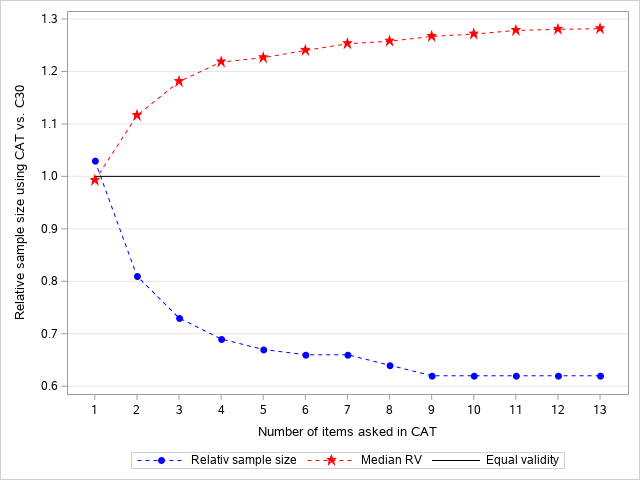

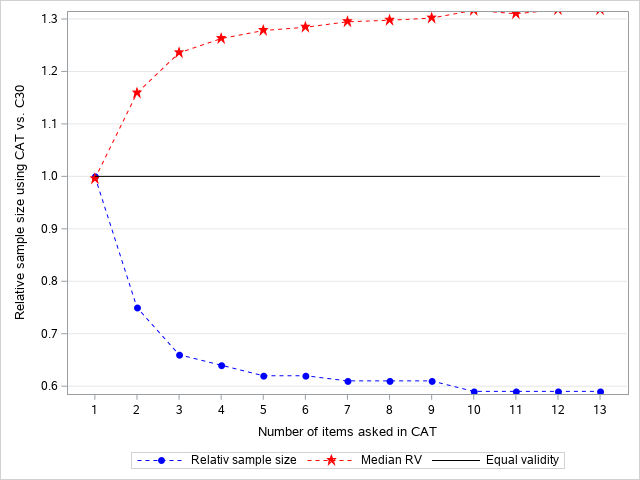


# Diarrhoea, fixed-precision CATs


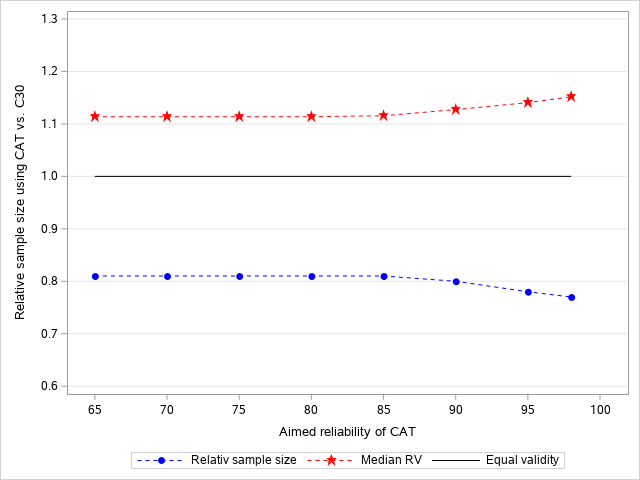


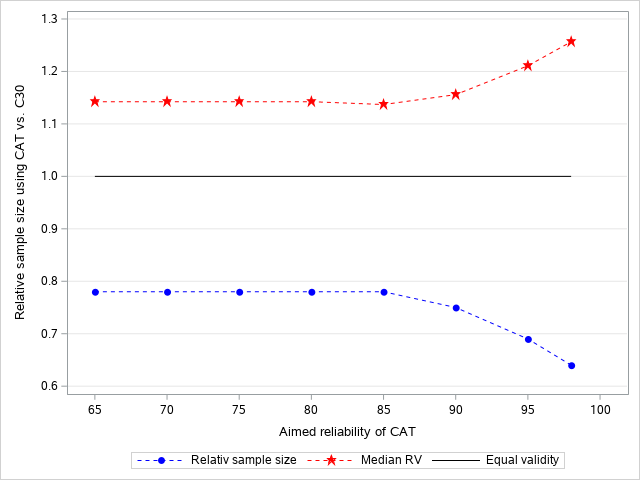

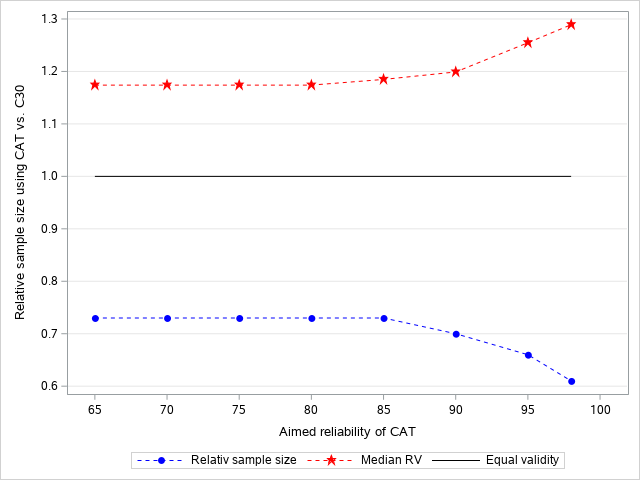


# Financial difficulties, fixed-length CATs


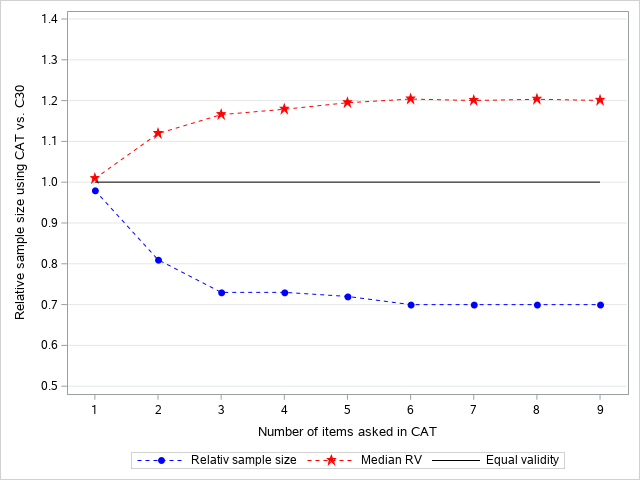


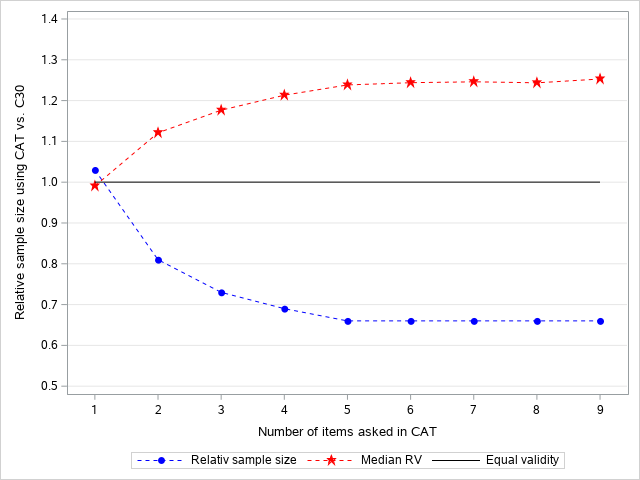

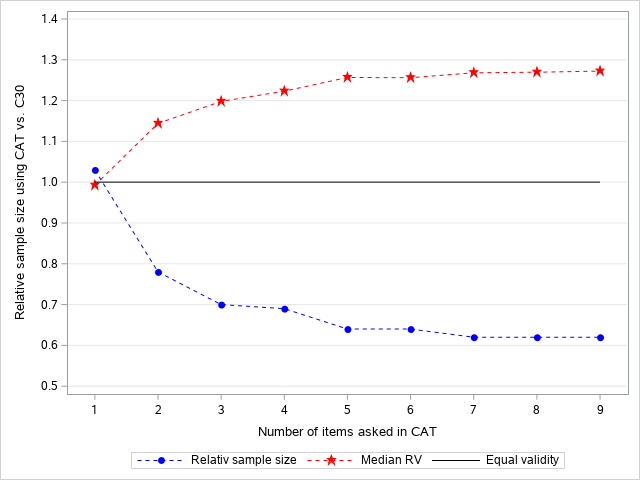


# Financial difficulties, fixed-precision CATs


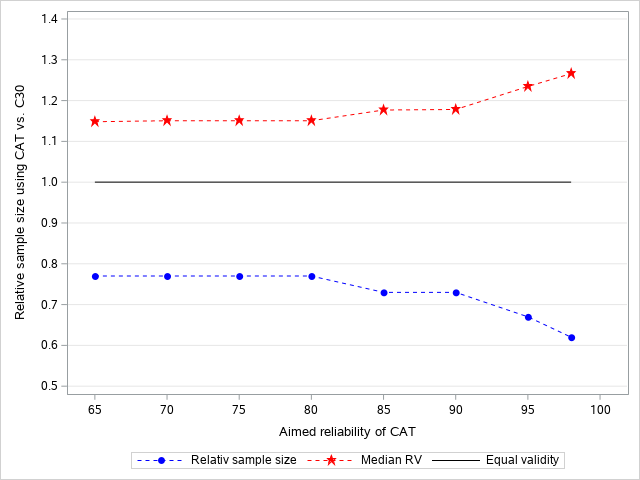


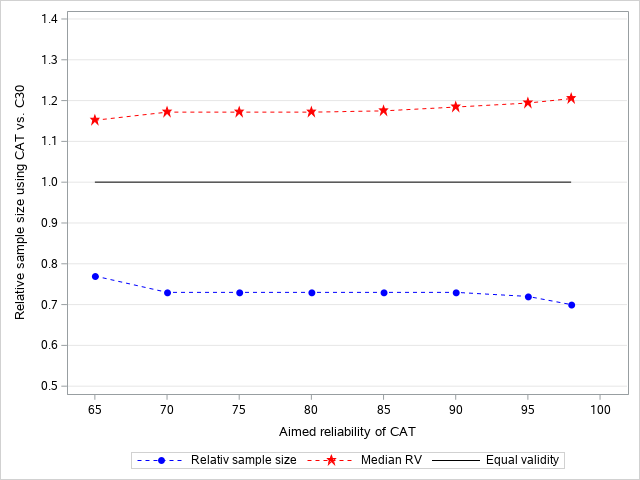

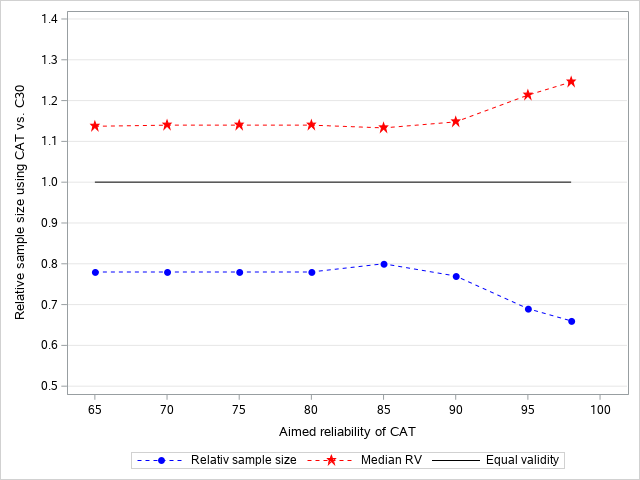


/************************************************************************************************
****The following code is provided “AS IS”, without warranty of any kind, either             ****
****expressed or implied. In no event shall the authors or copyright holders be liable for   ****
****any claims, damages or other liability arising from use of the code.                     ****
****If any questions, you are welcome to contact the author (below) but note that you are not**** 
****entitled to any hard copy documentation, maintenance, support, or updates of the code.   ****
****                                                                                         ****
****November 2023, Morten Aa. Petersen (Morten.Aagaard.Petersen@regionh.dk)                  ****
************************************************************************************************/


/************************************************************************************************
**The following code is for simulating CAT performance for the EORTC physical functioning      **
**item bank with 31 items. To use for other item banks the number of items etc.                **
**needs to be adjusted.                                                                        **
**                                                                                             **
**How to use:                                                                                  **
**Load the two datasets param and RV_SampleSavings and the macro CAT_sim into SAS by running   **
**the code below.                                                                              **
**Initiate the macro using:                                                                    **
**                                                                                             **
**CAT_sim(n_sim, n_grmin, n_grmax, pop_mean, pop_SD, pop, start_i, min_per_subd);              **
*where                                                                                         **
**n_sim:            Number of simulations                                                      **
**n_grmin:          Minimum N per group                                                        **
**n_grmax:          Maximum N per group                                                        **
**pop_mean:         Mean of population for reference group                                     **
**pop_SD:           SD of populations for both groups                                          **
**Pop:              Name of population for dataset, max 7 characters;                          **
**start_i:          Start item number;                                                         **
**min_per_subd:     Minimum number of items per subdomain;                                     **
**                                                                                             **
**Example:                                                                                     **
**%CAT_sim(1000, 50, 250, 49.2, 15.3, mild, 24, 1);                                            **
**                                                                                             **
**The macro produces data set RV_sim_results_PF_&pop with RVs etc. and                         **
**PF_sim_number_item_asked_&pop with number of items asked for fixed precision CATs            **
**                                                                                             **
**To minimise log output (which can be significant) possibly add:                              **
**options nosource nonotes;                                                                    **
**Revive log output by:                                                                        **
**options source notes;                                                                        **
************************************************************************************************/


*Item parameters; 
*Currently include example item parameter values. Add actual item parameters to use. 
*Use '.' for collapsed response categories;
**data** param; 
  input slope1 step1_1 - step1_3 
        slope2 step2_1 - step2_3
        slope3 step3_1 - step3_3 
        slope4 step4_1 - step4_3
        slope5 step5_1 - step5_3 
        slope6 step6_1 - step6_3 
        slope7 step7_1 - step7_3 
        slope8 step8_1 - step8_3 
        slope9 step9_1 - step9_3 
        slope10 step10_1 - step10_3 
        slope11 step11_1 - step11_3 
        slope12 step12_1 - step12_3 
        slope13 step13_1 - step13_3 
        slope14 step14_1 - step14_3 
        slope15 step15_1 - step15_3 
        slope16 step16_1 - step16_3 
        slope17 step17_1 - step17_3 
        slope18 step18_1 - step18_3 
        slope19 step19_1 - step19_3 
        slope20 step20_1 - step20_3 
        slope21 step21_1 - step21_3 
        slope22 step22_1 - step22_3 
        slope23 step23_1 - step23_3 
        slope24 step24_1 - step24_3 
        slope25 step25_1 - step25_3 
        slope26 step26_1 - step26_3 
        slope27 step27_1 - step27_3 
        slope28 step28_1 - step28_3 
        slope29 step29_1 - step29_3 
        slope30 step30_1 - step30_3 
        slope31 step31_1 - step31_3 
        subdomain_item1-subdomain_item31;
  datalines;  
        0.2 25 30 35 
        0.2 25 30 .
        0.2 25 . .
        0.2 25 30 35 
        0.2 25 30 .
        0.2 25 . .
        0.2 25 30 35 
        0.2 25 30 .
        0.2 25 . .
        0.2 25 30 35 
        0.2 25 30 .
        0.2 25 . .
        0.2 25 30 35 
        0.2 25 30 .
        0.2 25 . .
        0.2 25 30 35 
        0.2 25 30 .
        0.2 25 . .
        0.2 25 30 35 
        0.2 25 30 .
        0.2 25 . .
        0.2 25 30 35 
        0.2 25 30 .
        0.2 25 . .
        0.2 25 30 35 
        0.2 25 30 .
        0.2 25 . .
        0.2 25 30 35 
        0.2 25 30 .
        0.2 25 . .
        0.2 25 30 35 
        1 2 4 2 4 4 2 1 2 4 2 2 3 4 1 1 1 2 2 1 4 2 2 2 1 2 4 1 4 1 2
        ;
**run**;


*Relative validity (RV) to sample savings table. Used to calculate sample savings;
**data** RV_SampleSavings; 
  input RV_round sample_savings;
  datalines;  
        0.75 -77
        0.76 -72
        0.77 -72
        0.78 -64
        0.79 -59
        0.8 -56
        0.81 -52
        0.82 -48
        0.83 -45
        0.84 -41
        0.85 -38
        0.86 -34
        0.87 -31
        0.88 -30
        0.89 -27
        0.9 -23
        0.91 -20
        0.92 -19
        0.93 -16
        0.94 -14
        0.95 -11
        0.96 -9
        0.97 -6
        0.98 -5
        0.99 -3
        1 0
        1.01 2
        1.02 3
        1.03 5
        1.04 8
        1.05 9
        1.06 11
        1.07 12
        1.08 14
        1.09 16
        1.1 17
        1.11 19
        1.12 19
        1.13 20
        1.14 22
        1.15 23
        1.16 25
        1.17 27
        1.18 27
        1.19 28
        1.2 30
        1.21 31
        1.22 31
        1.23 33
        1.24 34
        1.25 34
        1.26 36
        1.27 38
        1.28 38
        1.29 39
        1.3 39
        1.31 41
        1.32 41
        1.33 42
        1.34 42
        1.35 44
        1.36 45
        1.37 45
        1.38 47
        1.39 47
        1.4 48
        1.41 48
        1.42 48
        1.43 50
        1.44 50
        1.45 52
        1.46 52
        1.47 52
        1.48 53
        1.49 53
        1.5 55
        100 .
        ;
**run**;


**************************SAS macro CAT_sim for simulation of CAT performance*****************************; 
*Relative validity (RV) and sample savings compared to QLQ-C30 scale in simulated data 
*with varying, random known mean differences for the true thetas;

**%MACRO** CAT_sim(n_sim, n_grmin, n_grmax, pop_mean, pop_SD, pop, start_i, min_per_subd);
data cat_sim_summary;
data cat_sim_totaldat;
data RV_sim_results;
run;

*Simulate data for two groups of random, equal size coming from populations with SD pop_SD and random different means;
%DO sim = **1** %TO &n_sim;
    data cat_sim_dat;
        sim_no=&sim;
        seed_number=**4326***sim_no;
        CALL STREAMINIT(seed_number); 
        *Random group size between selected min and max;
        n_gr=RAND('integer',&n_grmin, &n_grmax);
        n_sample=**2***n_gr;
        *Whether group to compare with has lower or higher mean;
        sign=RAND('integer',**0**, **1**);
        *Random effect size (ES=((m1-m2)/SD) of 0.2-0.5, defines mean difference of populations;
        ES=(**0.5**-**0.2**)*rand('uniform')+**0.2**;
        *Ensure group 1 has highest mean so always should get a positive t-test size;
        if sign=**0** then do;
            mean_gr1=&pop_mean;
            mean_gr2=&pop_mean-ES*&pop_SD;
        end;
        if sign=**1** then do;
            mean_gr2=&pop_mean;
            mean_gr1=&pop_mean+ES*&pop_SD;
        end;
        mean_diff=abs(mean_gr1-mean_gr2);
    run;

    *Generate data for simulating two groups of size n_gr;
    data cat_sim_dat;
    set  cat_sim_dat;
        sim_no=&sim;
        seed_number=**224***sim_no;
        CALL STREAMINIT(seed_number); 
        do i=**1** to n_gr;
            theta=rand('NORMAL',mean_gr1,&pop_SD);
            group=**1**;
            output;
        end;
        do i=**1** to n_gr;
            theta=rand('NORMAL',mean_gr2,&pop_SD);
            group=**2**;
            output;
        end;
    run;
    *Summarises the group means group sizes etc. of each simulation;
    data cat_sim_summary;
    set  cat_sim_summary cat_sim_dat;
    if sim_no ne **.**;
    run;

    *Generate random responses to all items based on the simulated theta scores;
    data cat_sim_dat2;
    set cat_sim_dat;
    if _n_ eq **1** then do;
        set param;
    end;

    data cat_sim_dat2;
    set  cat_sim_dat2;
    seed_number=**872***sim_no;
    CALL STREAMINIT(seed_number); 

    array slopes(**31**)   slope1-slope31;
    array steps(**93**)   step1_1-step1_3 step2_1-step2_3 step3_1-step3_3 step4_1-step4_3 step5_1-step5_3 step6_1-step6_3 step7_1-step7_3 step8_1-step8_3
                        step9_1-step9_3 step10_1-step10_3 step11_1-step11_3 step12_1-step12_3 step13_1-step13_3 step14_1-step14_3 step15_1-step15_3
                        step16_1-step16_3 step17_1-step17_3 step18_1-step18_3 step19_1-step19_3 step20_1-step20_3 step21_1-step21_3 step22_1-step22_3 
                        step23_1-step23_3 step24_1-step24_3 step25_1-step25_3 step26_1-step26_3 step27_1-step27_3 step28_1-step28_3 step29_1-step29_3 
                        step30_1-step30_3 step31_1-step31_3;
    array t(**3**)           t1-t3;
    array ps(**124**)       p1_0-p1_3 p2_0-p2_3 p3_0-p3_3 p4_0-p4_3 p5_0-p5_3 p6_0-p6_3 p7_0-p7_3 p8_0-p8_3
                        p9_0-p9_3 p10_0-p10_3 p11_0-p11_3 p12_0-p12_3 p13_0-p13_3 p14_0-p14_3 p15_0-p15_3 p16_0-p16_3
                        p17_0-p17_3 p18_0-p18_3 p19_0-p19_3 p20_0-p20_3 p21_0-p21_3 p22_0-p22_3 p23_0-p23_3 p24_0-p24_3
                        p25_0-p25_3 p26_0-p26_3 p27_0-p27_3 p28_0-p28_3 p29_0-p29_3 p30_0-p30_3 p31_0-p31_3; 
    array resp(**31**)       pf1-pf31; 
    array resp_NAA(**31**)   pfNAA1-pfNAA31; 
    array prob(**31**)       prob1-prob31;
    array cat_scores(**31**)       cat_theta1-cat_theta31; *theta scores after each step in CAT;
    array asked_cat_steps(**31**)   asked_cat_step1-asked_cat_step31; *step in CAT each item has been asked;
    array asked_items(**31**)       asked_item1-asked_item31; *Whether item has been asked only of relevance while running program;
    array item_inf(**31**)           inf_item1-inf_item31; *Inf of item in theta est of previous step;
    array cat_inf_step(**31**)       cat_inf_step1-cat_inf_step31; *total inf in theta est of current step after item asked in step;
    array item_inf_true(**31**)   inf_true_item1-inf_true_item31; *inf of each item in true theta score;
    array subdomain_items(**31**)   subdomain_item1-subdomain_item31;
    array subdomains(**4**)       subdomain1-subdomain4; 
    array cat_theta_p_rels(**8**)   cat_theta_p_rel65 cat_theta_p_rel70 cat_theta_p_rel75 cat_theta_p_rel80 cat_theta_p_rel85 
                                cat_theta_p_rel90 cat_theta_p_rel95 cat_theta_p_rel98;  * Estimates for precise fixed precision CAT;
    array cat_theta_q_rels(**8**)   cat_theta_q_rel65 cat_theta_q_rel70 cat_theta_q_rel75 cat_theta_q_rel80 cat_theta_q_rel85 
                                cat_theta_q_rel90 cat_theta_q_rel95 cat_theta_q_rel98;  * Estimates for quick fixed precision CAT;
    array cat_inf_p_rels(**8**)   cat_inf_p_rel65 cat_inf_p_rel70 cat_inf_p_rel75 cat_inf_p_rel80 cat_inf_p_rel85 
                                cat_inf_p_rel90 cat_inf_p_rel95 cat_inf_p_rel98;    *Information for precise fixed precision CAT;
    array cat_inf_q_rels(**8**)   cat_inf_q_rel65 cat_inf_q_rel70 cat_inf_q_rel75 cat_inf_q_rel80 cat_inf_q_rel85 
                                cat_inf_q_rel90 cat_inf_q_rel95 cat_inf_q_rel98;    *Information for quick fixed precision CAT;
    array fulfil_p_rels(**8**)       fulfil_p_rel65 fulfil_p_rel70 fulfil_p_rel75 fulfil_p_rel80 fulfil_p_rel85 
                                fulfil_p_rel90 fulfil_p_rel95 fulfil_p_rel98; *Whether precision fulfilled;
    array fulfil_q_rels(**8**)       fulfil_q_rel65 fulfil_q_rel70 fulfil_q_rel75 fulfil_q_rel80 fulfil_q_rel85 
                                fulfil_q_rel90 fulfil_q_rel95 fulfil_q_rel98; *Whether precision fulfilled;
    array items_asked_p_rels(**8**) items_asked_p_rel65 items_asked_p_rel70 items_asked_p_rel75 items_asked_p_rel80 items_asked_p_rel85 
                                items_asked_p_rel90 items_asked_p_rel95 items_asked_p_rel98; *number of items asked in precise fixed precision CATs;
    array items_asked_q_rels(**8**) items_asked_q_rel65 items_asked_q_rel70 items_asked_q_rel75 items_asked_q_rel80 items_asked_q_rel85 
                                items_asked_q_rel90 items_asked_q_rel95 items_asked_q_rel98; *number of items asked in quick fixed precision CATs;

    items_in_bank=**31**;       *number of items;

    do i=**1** to items_in_bank; 
        number_cat=**1**;
        *Calculation of the item response propabilities;
        do j=**1** to **3**; 
            if steps(j+**3***(i-**1**))=**.** then t(j)=**0**;
            if steps(j+**3***(i-**1**)) ne **.** then do;
                number_cat=number_cat+**1**; 
                if j=**1** then t(j)=exp(slopes(i)*(theta-steps(j+**3***(i-**1**))));
                if j>**1** then t(j)=t(j-**1**)*exp(slopes(i)*(theta-steps(j+**3***(i-**1**))));
            end;
        end;
        n=**1**+t1+t2+t3;
        ps(**4***(i-**1**)+**1**)=**1**/n; *'Very much';
        ps(**4***(i-**1**)+**2**)=t1/n;
        ps(**4***(i-**1**)+**3**)=t2/n;
        ps(**4***(i-**1**)+**4**)=t3/n; *'not at all';
        prob(i)=RAND('UNIFORM');
        *Select random response based on respopnse probabilities;
        if prob(i)<=**1**/n then resp(i)=**1**;
            else if prob(i)<=**1**/n+t1/n then resp(i)=**2**;
            else if prob(i)<=**1**/n+t1/n+t2/n then resp(i)=**3**;
            else if n ne **.** then resp(i)=**4**; 
    end;

    *C30 sum scale;
    PF_c30_sum=**100***(mean(pf5,pf13,pf15,pf19,pf24)-**1**)/**3**;

    *Recode categories for items with collapsed categories so maximum category (not at all) =4;
    do i=**1** to items_in_bank;
        resp_NAA(i)=resp(i); 
        if steps(**3***(i-**1**)+**3**)=**.** then resp_NAA(i)=resp(i)+**1**; *if two categories collapsed;
        if steps(**3***(i-**1**)+**2**)=**.** then resp_NAA(i)=resp(i)+**2**; *if three categories collapsed;
    end;

    ****************CAT simulation*****************;
    *Content coverage requirement: Number of items required per subdomain;
    min_per_subdomain=&min_per_subd;

    do i=**1** to **4**;   
        subdomains(i)=**0**;
    end;

    *A priori T-score mean and SD for caluculation of EAP score estimate and total information;
    apriori_mean=**47.143**;
    apriori_sd=**12.277**;

    **CAT step 1**;
    start_item=&start_i;

    *EAP theta estimate;
    irtxpxd=**0**;
    pxd=**0**;

    do quad=**0** to **100** by **0.2**;
        p=**1**;
        t0=**1**;
        do i=start_item to start_item; 
            do j=**1** to **3**; *Calculation of the item response propabilities;
                if steps(j+**3***(i-**1**))=**.** then t(j)=**0**;
                if steps(j+**3***(i-**1**)) ne **.** then do;
                    if j=**1** then t(j)=exp(slopes(i)*(quad-steps(j+**3***(i-**1**))));
                    if j>**1** then t(j)=t(j-**1**)*exp(slopes(i)*(quad-steps(j+**3***(i-**1**))));
                end;
            end;
            n=t0+t1+t2+t3;
            if resp(i)=**1** then p=p*t0/n;
            do k=**2** to **4**;
                if resp(i)=k then p=p*t(k-**1**)/n;
            end;
        end;
        d=pdf('normal',quad,apriori_mean,apriori_sd);
        irtxpxd=irtxpxd+quad*p*d;
        pxd=pxd+p*d;
    end;
    *EAP estimate;
    cat_scores(**1**)=irtxpxd/pxd;

    *start item has been asked;
    asked_items(start_item)=**1**;
    *Step in CAT the item is asked;
    asked_cat_steps(start_item)=**1**;
    *Asked 1 item from subdomain of start item;
    subdomains(subdomain_items(start_item))=subdomains(subdomain_items(start_item))+**1**; 
    *Information of start item in first estimate; 
    t1=**0**; t2=**0**; t3=**0**;
    if steps(**1**+**3***(start_item-**1**)) ne **.** then t1=exp(slopes(start_item)*(cat_scores(**1**)-steps(**1**+**3***(start_item-**1**))));
    if steps(**2**+**3***(start_item-**1**)) ne **.** then t2=t1*exp(slopes(start_item)*(cat_scores(**1**)-steps(**2**+**3***(start_item-**1**))));
    if steps(**3**+**3***(start_item-**1**)) ne **.** then t3=t2*exp(slopes(start_item)*(cat_scores(**1**)-steps(**3**+**3***(start_item-**1**))));
    tsum1=t1+**4***t2+**9***t3;
    tsum2=t1+**2***t2+**3***t3;
    n=**1**+t1+t2+t3;
    *Obtained information;
    cat_inf_step(**1**)=slopes(**1**)*slopes(**1**)*(tsum1/n-tsum2*tsum2/(n*n));

    **CAT steps 2 to max**;
    do cat_step=**2** to items_in_bank;
        *Estimation of information of each item in current theta estimate;
        max_inf_temp=**0**;
        do i=**1** to items_in_bank;
            if asked_items(i) ne **1** and 
                (subdomains(subdomain_items(i))<min_per_subdomain or min(subdomain1, subdomain2, subdomain3, subdomain4)>=min_per_subdomain) then do;
                t1=**0**; t2=**0**; t3=**0**;
                if steps(**1**+**3***(i-**1**)) ne **.** then t1=exp(slopes(i)*(cat_scores(cat_step-**1**)-steps(**1**+**3***(i-**1**))));
                if steps(**2**+**3***(i-**1**)) ne **.** then t2=t1*exp(slopes(i)*(cat_scores(cat_step-**1**)-steps(**2**+**3***(i-**1**))));
                if steps(**3**+**3***(i-**1**)) ne **.** then t3=t2*exp(slopes(i)*(cat_scores(cat_step-**1**)-steps(**3**+**3***(i-**1**))));
                tsum1=t1+**4***t2+**9***t3;
                tsum2=t1+**2***t2+**3***t3;
                n=**1**+t1+t2+t3;
                item_inf(i)=slopes(i)*slopes(i)*(tsum1/n-tsum2*tsum2/(n*n));
                if item_inf(i)>max_inf_temp then do;
                    max_inf_temp=item_inf(i);
                    ask_item=i;
                end;
            end;
        end;
        *Item selected in cat_step;
        asked_items(ask_item)=**1**;
        *Step in CAT the item is asked;
        asked_cat_steps(ask_item)=cat_step;
        *Count items asked from subdomain;
        subdomains(subdomain_items(ask_item))=subdomains(subdomain_items(ask_item))+**1**; 

        *EAP theta estimate;
        irtxpxd=**0**; 
        pxd=**0**;

        do quad=**0** to **100** by **0.2**;
            p=**1**; *initialize p when asking >1 item and need the combined propapility of the responses;
            t0=**1**;
            n=t0; *Initialize n to t0=1 so can check if some items have been answered;
            do i=**1** to items_in_bank;
                if asked_items(i)=**1** then do;
                    *Initiating values so are 0 if have collapsed UPPER categories;
                    t1=**0**; t2=**0**; t3=**0**;
                    if steps(**1**+**3***(i-**1**)) ne **.** then t1=exp(slopes(i)*(quad-steps(**1**+**3***(i-**1**))));
                    if steps(**2**+**3***(i-**1**)) ne **.** then t2=t1*exp(slopes(i)*(quad-steps(**2**+**3***(i-**1**))));
                    if steps(**3**+**3***(i-**1**)) ne **.** then t3=t2*exp(slopes(i)*(quad-steps(**3**+**3***(i-**1**))));
                    n=t0+t1+t2+t3;
                    if resp(i)=**1** then p_item=t0/n;
                    if resp(i)=**2** then p_item=t1/n;
                    if resp(i)=**3** then p_item=t2/n;
                    if resp(i)=**4** then p_item=t3/n;
                    p=p*p_item;
                end;
            end;
            if n>t0 then do; *n>t0 if at least 1 item has been answered;
                d=pdf('normal',quad,apriori_mean,apriori_sd); 
                irtxpxd=irtxpxd+quad*p*d;
                pxd=pxd+p*d;
            end;
        end;
        cat_scores(cat_step)=irtxpxd/pxd;

        *Total information of the asked items in the current theta estimate from above; 
        cat_inf_step(cat_step)=**0**;
        do i=**1** to items_in_bank;
            if asked_items(i)=**1** then do;
                t1=**0**; t2=**0**; t3=**0**;
                if steps(**1**+**3***(i-**1**)) ne **.** then t1=exp(slopes(i)*(cat_scores(cat_step)-steps(**1**+**3***(i-**1**))));
                if steps(**2**+**3***(i-**1**)) ne **.** then t2=t1*exp(slopes(i)*(cat_scores(cat_step)-steps(**2**+**3***(i-**1**))));
                if steps(**3**+**3***(i-**1**)) ne **.** then t3=t2*exp(slopes(i)*(cat_scores(cat_step)-steps(**3**+**3***(i-**1**))));
                tsum1=t1+**4***t2+**9***t3;
                tsum2=t1+**2***t2+**3***t3;
                n=**1**+t1+t2+t3;
                cat_inf_step(cat_step) = cat_inf_step(cat_step) + slopes(i)*slopes(i)*(tsum1/n-tsum2*tsum2/(n*n));
            end;
        end;

        ******************fixed precision CAT:***********************;
        *Quick CAT, ask max=8;
        quick_max=min(**8**,items_in_bank);
        if cat_step<=quick_max and min(subdomain1, subdomain2, subdomain3, subdomain4)>=min_per_subdomain then do;
            if (cat_inf_step(cat_step) + **1**/(apriori_sd*apriori_sd)) > **1**/((**1**-**0.65**)*apriori_sd*apriori_sd) and fulfil_q_rel65 ne **1** then do;
                fulfil_q_rel65=**1**;
                cat_theta_q_rel65=cat_scores(cat_step);
                cat_inf_q_rel65=cat_inf_step(cat_step);
                items_asked_q_rel65=cat_step;
            end;
            if (cat_inf_step(cat_step) + **1**/(apriori_sd*apriori_sd)) > **1**/((**1**-**0.70**)*apriori_sd*apriori_sd) and fulfil_q_rel70 ne **1** then do;
                fulfil_q_rel70=**1**;
                cat_theta_q_rel70=cat_scores(cat_step);
                cat_inf_q_rel70=cat_inf_step(cat_step);
                items_asked_q_rel70=cat_step;
            end;
            if (cat_inf_step(cat_step) + **1**/(apriori_sd*apriori_sd)) > **1**/((**1**-**0.75**)*apriori_sd*apriori_sd) and fulfil_q_rel75 ne **1** then do;
                fulfil_q_rel75=**1**;
                cat_theta_q_rel75=cat_scores(cat_step);
                cat_inf_q_rel75=cat_inf_step(cat_step);
                items_asked_q_rel75=cat_step;
            end;
            if (cat_inf_step(cat_step) + **1**/(apriori_sd*apriori_sd)) > **1**/((**1**-**0.80**)*apriori_sd*apriori_sd) and fulfil_q_rel80 ne **1** then do;
                fulfil_q_rel80=**1**;
                cat_theta_q_rel80=cat_scores(cat_step);
                cat_inf_q_rel80=cat_inf_step(cat_step);
                items_asked_q_rel80=cat_step;
            end;
            if (cat_inf_step(cat_step) + **1**/(apriori_sd*apriori_sd)) > **1**/((**1**-**0.85**)*apriori_sd*apriori_sd) and fulfil_q_rel85 ne **1** then do;
                fulfil_q_rel85=**1**;
                cat_theta_q_rel85=cat_scores(cat_step);
                cat_inf_q_rel85=cat_inf_step(cat_step);
                items_asked_q_rel85=cat_step;
            end;
            if (cat_inf_step(cat_step) + **1**/(apriori_sd*apriori_sd)) > **1**/((**1**-**0.90**)*apriori_sd*apriori_sd) and fulfil_q_rel90 ne **1** then do;
                fulfil_q_rel90=**1**;
                cat_theta_q_rel90=cat_scores(cat_step);
                cat_inf_q_rel90=cat_inf_step(cat_step);
                items_asked_q_rel90=cat_step;
            end;
            if (cat_inf_step(cat_step) + **1**/(apriori_sd*apriori_sd)) > **1**/((**1**-**0.95**)*apriori_sd*apriori_sd) and fulfil_q_rel95 ne **1** then do;
                fulfil_q_rel95=**1**;
                cat_theta_q_rel95=cat_scores(cat_step);
                cat_inf_q_rel95=cat_inf_step(cat_step);
                items_asked_q_rel95=cat_step;
            end;
            if (cat_inf_step(cat_step) + **1**/(apriori_sd*apriori_sd)) > **1**/((**1**-**0.98**)*apriori_sd*apriori_sd) and fulfil_q_rel98 ne **1** then do;
                fulfil_q_rel98=**1**;
                cat_theta_q_rel98=cat_scores(cat_step);
                cat_inf_q_rel98=cat_inf_step(cat_step);
                items_asked_q_rel98=cat_step;
            end;
            *If reliability criterion not fulfilled then use score and information in 8th step;
            if cat_step=quick_max then do;
                do j=**1** to **8**;
                    if fulfil_q_rels(j) ne **1** then do;
                        cat_theta_q_rels(j)=cat_scores(cat_step);
                        cat_inf_q_rels(j)=cat_inf_step(cat_step);
                        items_asked_q_rels(j)=cat_step;
                    end;
                end;
            end;
        end;

        *Precise CAT, ask max=12;
        precise_max=min(**12**,items_in_bank);
        if cat_step<=precise_max and min(subdomain1, subdomain2, subdomain3, subdomain4)>=min_per_subdomain then do;
            if (cat_inf_step(cat_step) + **1**/(apriori_sd*apriori_sd)) > **1**/((**1**-**0.65**)*apriori_sd*apriori_sd) and fulfil_p_rel65 ne **1** then do;
                fulfil_p_rel65=**1**;
                cat_theta_p_rel65=cat_scores(cat_step);
                cat_inf_p_rel65=cat_inf_step(cat_step);
                items_asked_p_rel65=cat_step;
            end;
            if (cat_inf_step(cat_step) + **1**/(apriori_sd*apriori_sd)) > **1**/((**1**-**0.70**)*apriori_sd*apriori_sd) and fulfil_p_rel70 ne **1** then do;
                fulfil_p_rel70=**1**;
                cat_theta_p_rel70=cat_scores(cat_step);
                cat_inf_p_rel70=cat_inf_step(cat_step);
                items_asked_p_rel70=cat_step;
            end;
            if (cat_inf_step(cat_step) + **1**/(apriori_sd*apriori_sd)) > **1**/((**1**-**0.75**)*apriori_sd*apriori_sd) and fulfil_p_rel75 ne **1** then do;
                fulfil_p_rel75=**1**;
                cat_theta_p_rel75=cat_scores(cat_step);
                cat_inf_p_rel75=cat_inf_step(cat_step);
                items_asked_p_rel75=cat_step;
            end;
            if (cat_inf_step(cat_step) + **1**/(apriori_sd*apriori_sd)) > **1**/((**1**-**0.80**)*apriori_sd*apriori_sd) and fulfil_p_rel80 ne **1** then do;
                fulfil_p_rel80=**1**;
                cat_theta_p_rel80=cat_scores(cat_step);
                cat_inf_p_rel80=cat_inf_step(cat_step);
                items_asked_p_rel80=cat_step;
            end;
            if (cat_inf_step(cat_step) + **1**/(apriori_sd*apriori_sd)) > **1**/((**1**-**0.85**)*apriori_sd*apriori_sd) and fulfil_p_rel85 ne **1** then do;
                fulfil_p_rel85=**1**;
                cat_theta_p_rel85=cat_scores(cat_step);
                cat_inf_p_rel85=cat_inf_step(cat_step);
                items_asked_p_rel85=cat_step;
            end;
            if (cat_inf_step(cat_step) + **1**/(apriori_sd*apriori_sd)) > **1**/((**1**-**0.90**)*apriori_sd*apriori_sd) and fulfil_p_rel90 ne **1** then do;
                fulfil_p_rel90=**1**;
                cat_theta_p_rel90=cat_scores(cat_step);
                cat_inf_p_rel90=cat_inf_step(cat_step);
                items_asked_p_rel90=cat_step;
            end;
            if (cat_inf_step(cat_step) + **1**/(apriori_sd*apriori_sd)) > **1**/((**1**-**0.95**)*apriori_sd*apriori_sd) and fulfil_p_rel95 ne **1** then do;
                fulfil_p_rel95=**1**;
                cat_theta_p_rel95=cat_scores(cat_step);
                cat_inf_p_rel95=cat_inf_step(cat_step);
                items_asked_p_rel95=cat_step;
            end;
            if (cat_inf_step(cat_step) + **1**/(apriori_sd*apriori_sd)) > **1**/((**1**-**0.98**)*apriori_sd*apriori_sd) and fulfil_p_rel98 ne **1** then do;
                fulfil_p_rel98=**1**;
                cat_theta_p_rel98=cat_scores(cat_step);
                cat_inf_p_rel98=cat_inf_step(cat_step);
                items_asked_p_rel98=cat_step;
            end;
            *If reliability criterion not fulfilled then use score and information in 12th step;
            if cat_step=precise_max then do;
                do j=**1** to **8**;
                    if fulfil_p_rels(j) ne **1** then do;
                        cat_theta_p_rels(j)=cat_scores(cat_step);
                        cat_inf_p_rels(j)=cat_inf_step(cat_step);
                        items_asked_p_rels(j)=cat_step;
                    end;
                end;
            end;
        end;
    end;

    drop i j n number_cat d irtxpxd pxd n t0-t3 quad tsum1 tsum2 cat_step;
    run;

    *Total dataset comprising all simulated responses, scores etc.;
    data cat_sim_totaldat;
    set  cat_sim_totaldat cat_sim_dat2;
    if sim_no ne **.**;
    run;
    
    *T-test of group difference;
    ods graphics off;
    ods exclude all;
    proc ttest data=cat_sim_dat2;   
        class group;
        var PF_c30_sum cat_theta1-cat_theta31 cat_theta_p_rel65 cat_theta_p_rel70 cat_theta_p_rel75 cat_theta_p_rel80 cat_theta_p_rel85
            cat_theta_p_rel90 cat_theta_p_rel95 cat_theta_p_rel98 cat_theta_q_rel65 cat_theta_q_rel70 cat_theta_q_rel75 cat_theta_q_rel80 cat_theta_q_rel85
            cat_theta_q_rel90 cat_theta_q_rel95 cat_theta_q_rel98 theta;
        ods output statistics=sout Ttests=tout;
    run;
    ods graphics on;
    ods exclude none;
    
    data tout2;
    set  tout;
    if method='Pooled';
    run;
    data tout2;
    set  tout2;
    if _N_=**1** then type='SUM';
    else type='CAT';
    run;
    data tout_cat;
    set  tout2;
    if type='CAT';
    number=**1**;
    rename variable=cat_score tvalue=tvalue_cat df=df_cat probt=p_cat;
    data tout_SUM;
    set  tout2;
    if type='SUM';
    number=**1**;
    rename variable=var_sum tvalue=tvalue_sum df=df_sum probt=p_sum;
    run;
    data ttest;
    merge tout_cat tout_SUM;
    by number;
    rv=tvalue_cat/tvalue_sum;
    *To avoid both test are in wrong direction and the one 'most wrong' results in large RV;
    if tvalue_cat<**0** and tvalue_sum<**0** then rv=**.**;
    *To avaoid negative RVs but still use tests subtract 2*negative test from both tests so still have same difference but 
    just moves tests to 'positive side'. If deleting these tests would be unfair to test that was positive and similarly if just reverse sign;
    if tvalue_cat<**0** and tvalue_sum>**0** then rv=-tvalue_cat/(tvalue_sum-**2***tvalue_cat);
    if tvalue_cat>**0** and tvalue_sum<**0** then rv=-(tvalue_cat-**2***tvalue_sum)/tvalue_sum;

    if rv>**1** then RV_above_1=**1**;
    if **0**<=rv<=**1** then RV_above_1=**0**;
    sim_no=&sim;
    drop type number;
    run;

    data RV_sim_results;
    set RV_sim_results ttest;
    if cat_score='' then delete;
    run;    

%END;

data RV_sim_results_PF_&pop;
merge RV_sim_results cat_sim_summary;
by sim_no;
run;

title 'Average simulated group size, effect size, and group difference';
proc means data=RV_sim_results_PF_&pop n mean stderr median min max maxdec=**2**;
    var n_gr ES mean_diff;
run;
title;

proc means data=RV_sim_results_PF_&pop n mean stderr median min max maxdec=**2** noprint;
    class cat_score;
    var rv;
output out=Rv_medians n=n mean=mean_rv median=median_rv;
run;

DATA Rv_medians;
set  Rv_medians;
if cat_score ne '';
run;
DATA Rv_medians;
set  Rv_medians;
sd_mean_est=**1**/mean_rv;
sd_median_est=**1**/median_rv;
if **1**<=_N_<=**31** then cat_step=input(substr(cat_score,**10**), **3.**); 
else cat_step=input(substr(cat_score,**16**), **3.**);
fixed_precision_type=substr(cat_score,**11**,**1**);
run;
proc sort data=Rv_medians;
    by cat_step;
run;

data Rv_medians2;
set  Rv_medians;
*Use median RVs to reduce effect of extrem\outlier RVs;
rv_round=round(median_rv,**0.01**);
*Sample savings for RVs outside 0.75-1.50 are calculated from 'RV to sample savings' fitted regression curve;
*Identify all RVs outside 0.75-1.50 with value 100;
if **0**<rv_round<**0.75** or rv_round>**1.50** then rv_round=**100.00**;
if cat_step ne **.**;
drop _type_ _freq_;
run;
proc sort data=Rv_medians2;
    by rv_round;
run;
data Rv_medians3;
merge Rv_medians2 RV_SampleSavings;
by rv_round;

*Estimated savings for RVs outside 0.75-1.50;
if rv_round = **100** then sample_savings=round(**100***(-**0.664***median_rv****4**+**4.93***median_rv****3**-**13.75***median_rv****2**+**17.42***median_rv-**7.95**),**1**);
relativ_sample_size=(**100**-sample_savings)/**100**;
relativ_savings=**100***(**1**-relativ_sample_size);
if cat_score ne '';
label relativ_sample_size='Relative sample size';
label median_rv='Median RV';
run;

proc sort data=Rv_medians3;
    by cat_step;
run;

title 'Median relative validities (RV) and sample savings with different CATs compared to C30 scale';
proc print data=Rv_medians3;
    var cat_score n median_rv sample_savings; 
    where cat_score ne 'theta';
run;

title 'Number of items asked in fixed precision CATs';
proc means data=cat_sim_totaldat n mean median p5 p25 p75 p95 min max maxdec=**3**;
    var items_asked_q_rel65 items_asked_q_rel70 items_asked_q_rel75 items_asked_q_rel80 items_asked_q_rel85 
        items_asked_q_rel90 items_asked_q_rel95 items_asked_q_rel98
        items_asked_p_rel65 items_asked_p_rel70 items_asked_p_rel75 items_asked_p_rel80 items_asked_p_rel85 
        items_asked_p_rel90 items_asked_p_rel95 items_asked_p_rel98;
    output out=PF_sim_number_item_asked_&pop mean=  items_asked_q_rel65 items_asked_q_rel70 items_asked_q_rel75 items_asked_q_rel80 items_asked_q_rel85 
                                                    items_asked_q_rel90 items_asked_q_rel95 items_asked_q_rel98
                                                    items_asked_p_rel65 items_asked_p_rel70 items_asked_p_rel75 items_asked_p_rel80 items_asked_p_rel85 
                                                    items_asked_p_rel90 items_asked_p_rel95 items_asked_p_rel98;
run;
data PF_sim_number_item_asked_&pop;
set  PF_sim_number_item_asked_&pop;
drop _freq_ _type_;
run;

*Actual reliability for variable length CATs;
data cat_sim_totaldat;
set  cat_sim_totaldat;
apriori_sd=**12.277**; 
array rel_actual(**16**) rel_actual_q_rel65 rel_actual_q_rel70 rel_actual_q_rel75 rel_actual_q_rel80 rel_actual_q_rel85 
                     rel_actual_q_rel90 rel_actual_q_rel95 rel_actual_q_rel98
                     rel_actual_p_rel65 rel_actual_p_rel70 rel_actual_p_rel75 rel_actual_p_rel80 rel_actual_p_rel85 
                     rel_actual_p_rel90 rel_actual_p_rel95 rel_actual_p_rel98;
array inf(**16**)        cat_inf_q_rel65 cat_inf_q_rel70 cat_inf_q_rel75 cat_inf_q_rel80 cat_inf_q_rel85 
                     cat_inf_q_rel90 cat_inf_q_rel95 cat_inf_q_rel98
                     cat_inf_p_rel65 cat_inf_p_rel70 cat_inf_p_rel75 cat_inf_p_rel80 cat_inf_p_rel85 
                     cat_inf_p_rel90 cat_inf_p_rel95 cat_inf_p_rel98;
do i = **1** to **16**;
    rel_actual(i)=**1**-((**1**/(inf(i)+**1**/(apriori_sd*apriori_sd)))/(apriori_sd*apriori_sd));
end;
run;

title 'Actually obtained reliability with fixed precision CATs';
proc means data=cat_sim_totaldat n mean std median p5 p25 p75 p95 min max maxdec=**4**;
    var rel_actual_q_rel65 rel_actual_q_rel70 rel_actual_q_rel75 rel_actual_q_rel80 
        rel_actual_q_rel85 rel_actual_q_rel90 rel_actual_q_rel95 rel_actual_q_rel98
        rel_actual_p_rel65 rel_actual_p_rel70 rel_actual_p_rel75 rel_actual_p_rel80 
        rel_actual_p_rel85 rel_actual_p_rel90 rel_actual_p_rel95 rel_actual_p_rel98;
run;
title;

**%MEND** CAT_sim;
